# RNA export factor Ddx19 is required for nuclear import of the SRF coactivator MKL1

**DOI:** 10.1038/ncomms6978

**Published:** 2015-01-14

**Authors:** Eeva Kaisa Rajakylä, Tiina Viita, Salla Kyheröinen, Guillaume Huet, Richard Treisman, Maria K. Vartiainen

**Affiliations:** 1 Program in Cell and Molecular Biology, Institute of Biotechnology, University of Helsinki, Viikinkaari 9, Helsinki 00014, Finland; 2 Transcription group, Cancer Research UK London Research Institute, London WC2A 3LY, UK

## Abstract

Controlled transport of macromolecules between the cytoplasm and nucleus is essential for homeostatic regulation of cellular functions. For instance, gene expression entails coordinated nuclear import of transcriptional regulators to activate transcription and nuclear export of the resulting messenger RNAs for cytoplasmic translation. Here we link these two processes by reporting a novel role for the mRNA export factor Ddx19/Dbp5 in nuclear import of MKL1, the signal-responsive transcriptional activator of SRF. We show that Ddx19 is not a general nuclear import factor, and that its specific effect on MKL1 nuclear import is separate from its role in mRNA export. Both helicase and nuclear pore-binding activities of Ddx19 are dispensable for MKL1 nuclear import, but RNA binding is required. Mechanistically, Ddx19 operates by modulating the conformation of MKL1, which affects its interaction with Importin-β for efficient nuclear import. Thus, Ddx19 participates in mRNA export, translation and nuclear import of a key transcriptional regulator.

## Abstract (web summary)

Nuclear import of transcriptional regulators and export of mRNA are essential steps for the manufacture of proteins in the cytoplasm. Here, Rajakylä *et al.* link these two activities by showing that the mRNA export factor Ddx19 promotes the nuclear import of the transcriptional coactivator MKL1.

## Graphical abstract

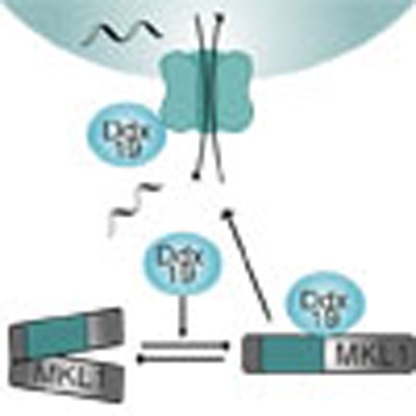

Communication between the two main compartments of the cell, the cytoplasm and the nucleus, is essential for maintaining cellular homeostasis and for the ability to respond to changing circumstances. For example, cytoplasmic signalling pathways must impinge on nuclear gene expression machineries to elicit specific transcriptional programmes and the resulting mRNA molecules need to be transported to the cytoplasm for translation. Some of the produced proteins will then be transported back to the nucleus, to act, for example, as components of the genome maintenance and gene expression machineries. Coordination of nuclear import and export events of different macromolecules, for example, proteins and RNA, is therefore at the heart of many cell biological processes^1^.

Nuclear pore complexes (NPCs) create semi-permeable channels across the nuclear envelope and mediate the selective transport of macromolecules between the cytoplasm and the nucleus. Although NPCs are freely permeable to ions, water and, for example, proteins smaller than ~40  kDa, larger proteins require an active, energy-dependent mechanism that includes soluble nuclear transport factors, most often karyopherins (Kaps) and the small GTPase Ran to control the directionality of the transport. Kaps recognize transport signals that guide their cargo either to the nucleus (nuclear localization signal; NLS) or out of the nucleus (nuclear export signal; NES)^2^,^3^. Import complexes are dissociated by RanGTP binding in the nucleus, whereas export complexes are formed via RanGTP association^4^. A classic example of Kap-mediated transport is the nucleo-cytoplasmic shuttling of the transcriptional coactivator Megakaryoplastic leukemia 1 protein (MKL1; also known as MAL or MRTF-A)^5^,^6^.

MKL1 is an actin-binding coactivator that mediates the signals from cellular G-actin levels to the essential transcription factor serum response factor (SRF)^7^,^8^,^9^. Together, these transcriptional regulators control the expression of target genes encoding proteins that are components of the actin cytoskeleton and, therefore, many important biological processes such as development, acto-myosin activity and cell–extracellular matrix adhesions are dependent on them^10^. Nucleo-cytoplasmic shuttling of MKL1 is central in its role as a transcriptional coactivator. In unstimulated cells, MKL1 is mainly cytoplasmic and it accumulates in the nucleus on signals that induce actin polymerization and thus decreased G-actin levels^7^. Actin regulates both nuclear import and export of MKL1, as well as its activity within the nucleus^5^. The amino-terminal RPEL domain of MKL1 is sufficient to mediate its nucleo-cytoplasmic shuttling, because it includes the actin-binding motifs, the NLS^5^,^6^,^7^,^11^ and the NES^12^. MKL1 contains an unusually long bipartite NLS that is recognized by the Importin-α/β (Ipoα/β) heterodimer. Ipoα/β and actin compete for binding to MKL1 RPEL domain^6^ and structural studies have shown that actin sterically occludes the NLS, preventing its recognition by Ipoα/β heterodimer^13^. Nuclear export of MKL1 is mediated by Crm1/exportin-1 and actin-binding is required for efficient nuclear export^5^. Sequences within both the RPEL and the glutamine-rich (Q) domain of MKL1 have been implicated as leucine-rich Crm1-binding sites^12^, but the molecular mechanisms, and especially the contribution of actin, warrants further investigations.

Nuclear export of mRNA begins with the packaging of the processed pre-mRNA into messenger ribonucleoprotein (mRNP) complexes that are targeted to the NPCs. Unlike protein transport, mRNA export is not dependent on Kaps and only indirectly dependent on the Ran gradient. Instead, the heterodimer of Nxf1 and Nxt1 act as the transport factors and the directional passage of mRNAs is generated by Ddx19 (better known as Dbp5 in yeast), Gle1 and inositol hexakisphosphate (IP_6_). Ddx19 belongs to the DEAD-box protein family that is capable of ATP-dependent remodelling of RNA/protein complexes^14^. Although Ddx19 is known to shuttle between the nucleus and cytoplasm in a Crm1-dependent manner^15^, it is primarily located in the cytoplasm and enriched at the nuclear envelope, specifically at the cytoplasmic filaments of the NPC^15^,^16^. Combination of biochemical, structural and genetic studies mainly in yeast have yielded comprehensive models of how Ddx19 functions in mRNA export^17^. Binding of Gle1-IP_6_ enhances the ATP loading of Ddx19, and thus its binding to the RNA in the mRNP complex. This then stimulates both the release of Gle1-IP_6_ and the ATPase activity of Ddx19. ATP hydrolysis leads to a conformation change in Ddx19, resulting in remodelling of the mRNP composition and release of mRNA from Ddx19 (refs 18, 19). Structural studies, on the other hand, have suggested that Gle1-IP_6_ would act together with Nup159 to stabilize a Dbp5 conformation that cannot bind RNA, and thereby promote RNA release^20^. Moreover, other mechanisms such as phosphorylation-based regulation of Npl3, an SR-like protein, by Glc7 is required for mRNA export by promoting the association of Mex67p/Nxf1 with mRNA^21^. In addition to its role in nuclear export, the yeast Dbp5 has been linked to translation termination. Interestingly, also in this context Dbp5 seems to function together with Gle1 and IP_6_, and it has been proposed that Dbp5 acts to remodel the RNA/protein complex in the ribosome to allow efficient stop codon recognition^22^. Ddx19 can therefore control the fate of the mRNA all the way from the NPC to the ribosome.

In this study we propose a novel and specific role for Ddx19 in nuclear import of MKL1 and, as such, in regulation of SRF-mediated gene transcription. RNA-binding but neither helicase nor Nup214-binding activities of Ddx19 are required for MKL1 nuclear import. We further show that Ddx19 is required for efficient binding of Ipoβ to MKL1, and demonstrate that Ddx19 regulates the conformation of MKL1 *in vivo*. We therefore speculate that Ddx19 binding to MKL1 induces an open conformation, which is prerequisite for efficient NLS recognition by the nuclear import complex. Our study therefore adds an additional layer to MKL1-SRF regulation.

## Results

### Ddx19 specifically regulates the nuclear import of MKL1

**Figure 1:**
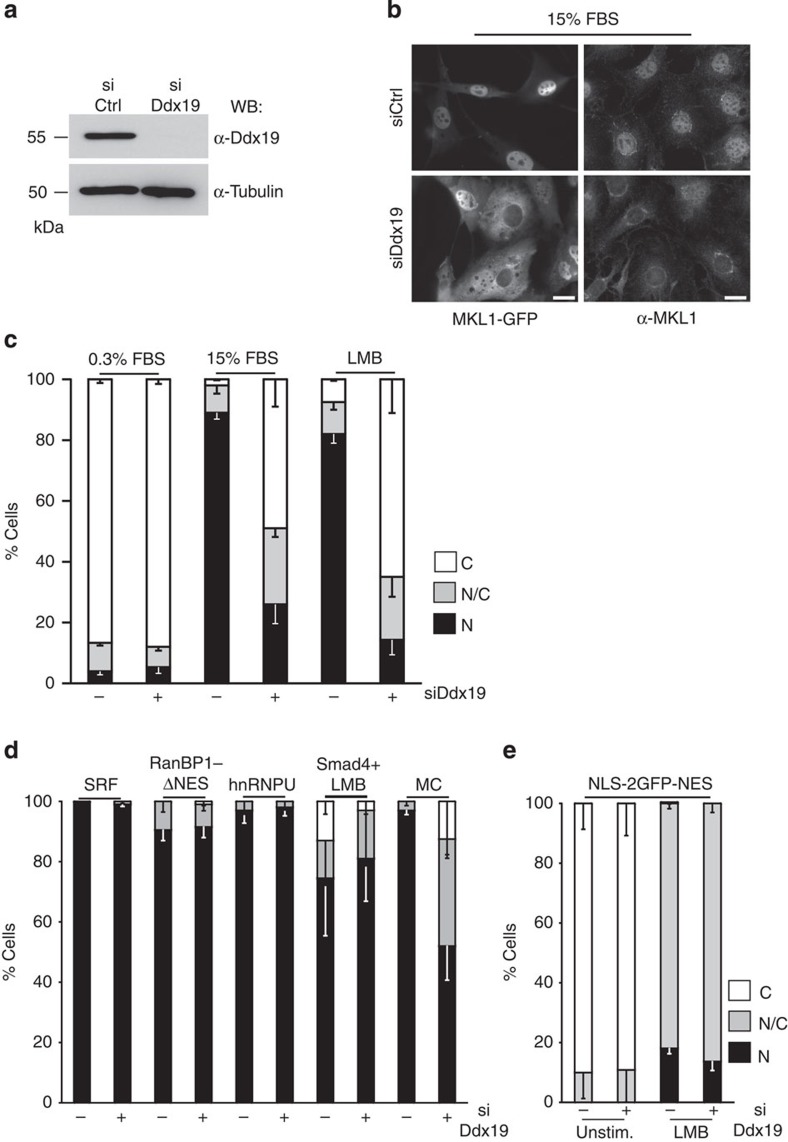
Ddx19 is specifically required for the nuclear localization of MKL1. (**a**) Western blotting (WB) of cells treated with control (Ctrl) or Ddx19 siRNAs (si). (**b**) Localization of MKL1-GFP (left panels) and endogenous MKL1 (α-MKL1; right panel) in serum (FBS)-stimulated NIH 3T3 cells. Scale bars, 20 μm. (**c**) Quantification of MKL1-GFP localization in serum-starved (0.3% FBS), serum-stimulated (15% FBS) and LMB-treated control (−) or Ddx19 (+) siRNA-transfected cells. C, cytoplasmic; N/C, pancellular; N, nuclear. One hundred cells per point; *n*=3; error bars are s.e.m. (**d**) Localization of nuclear proteins in NIH 3T3 cells transfected with control (−) or Ddx19 (+) siRNA. SRF (serum response factor), RanBPI-ΔNES (Ran binding protein 1 lacking nuclear export signal), hnRNPU (heterogeneous nuclear ribonucleoprotein U), Smad4+LMB (Smad4 stimulated with leptomycin B), MC (myocardin). More than fifty cells per point; *n*=2; error bars are s.d. (**e**) Localization of NLS-2GFP-NES in serum-starved (0.3% FBS) or LMB-treated cells transfected with control (−) or Ddx19 (+) siRNAs. One hundred cells per point; *n*=2; error bars are s.d.

We previously showed that nuclear import of MKL1 is dependent on the classical nuclear import pathway mediated by Ipoα/β-heterodimer and regulated by actin^6^,^13^. To investigate whether additional pathways are involved in nuclear localization of this important transcription coactivator, we screened a library of small interfering RNAs (siRNAs) directed against nuclear import and export components for effects on MKL1 nuclear localization. To our surprise, we found that in addition to Ipoβ, serum-induced nuclear accumulation of MKL1 was inhibited by RNA interference (RNAi)-mediated depletion of Ddx19 (Fig. 1a–c), a DEAD-box helicase with reported functions in mRNA export and translation^16^,^22^,^23^, in the mouse embryonic fibroblast cell line NIH 3T3, where the MKL1-SRF pathway has been extensively studied^5^,^7^,^24^. In these cells, MKL1 localizes to the cytoplasm in unstimulated conditions, but accumulates in the nucleus on stimulations, such as serum, which polymerize actin (Fig. 1c and Supplementary Fig. 1a), as shown before^5^,^7^. Depletion of Ddx19 prevented the serum-induced nuclear accumulation of both endogenous MKL1 and MKL1-GFP (Fig. 1b). Significantly, Ddx19 depletion also inhibited nuclear accumulation of MKL1 on Leptomycin B (LMB) treatment (Fig. 1c and Supplementary Fig. 1a), which inactivates Crm1/Exportin-1 (ref. 25), the established nuclear export receptor for MKL1 (ref. 5). As LMB affects only nuclear export, this result demonstrates that Ddx19 specifically impinges on nuclear import of MKL1. The efficient depletion of Ddx19 was confirmed by western blotting (Fig. 1a) and fluorescence *in situ* hybridization assay, where depletion of Ddx19 resulted in nuclear mRNA retention (Supplementary Fig. 1b,c). Depletion of Ddx19 prevented the nuclear accumulation of MKL1 also in the human MCF7 cell line (Supplementary Fig. 1d,e). Finally, the Ddx19-depletion phenotype was also significantly rescued by overexpressing siRNA-resistant version of the protein (Fig. 3b,c), confirming that the defect in MKL1 nuclear localization was specifically due to Ddx19 depletion.

To our knowledge, Ddx19 has not been linked to nuclear import before. However, it is well-established that Ddx19 operates at the NPC to facilitate nuclear export of mRNAs^16^,^18^,^19^,^23^ and its depletion could therefore lead to a general defect in NPC function. To study this possibility, we next monitored the localization of a set of control proteins in Ddx19-depleted cells. These proteins included the constitutively nuclearly localized SRF, Ran-binding protein 1 lacking NES and hnRNP-U. Smad4, on the other hand, shuttles in and out of the nucleus and accumulates in the nucleus on LMB treatment^26^, similar to NLS-2GFP-NES construct, which uses exactly the same nuclear import and export factors, Ipoα/β and Crm1 (refs 5, 6), respectively, as MKL1 does. Depletion of Ddx19 did not cause a defect in the nuclear localization of any of these proteins (Fig. 1d,e and Supplementary Fig. 2a,b), demonstrating that depletion of Ddx19 does not cause a general block in nuclear import. However, nuclear localization of another MRTF family member, myocardin, which localizes to the nucleus in unstimulated cells^11^, was significantly impaired in Ddx19-depleted cells (Fig. 1d and Supplementary Fig. 2a). Therefore, MRTF family members seem to share the requirement for Ddx19 in their nuclear import.

**Figure 2:**
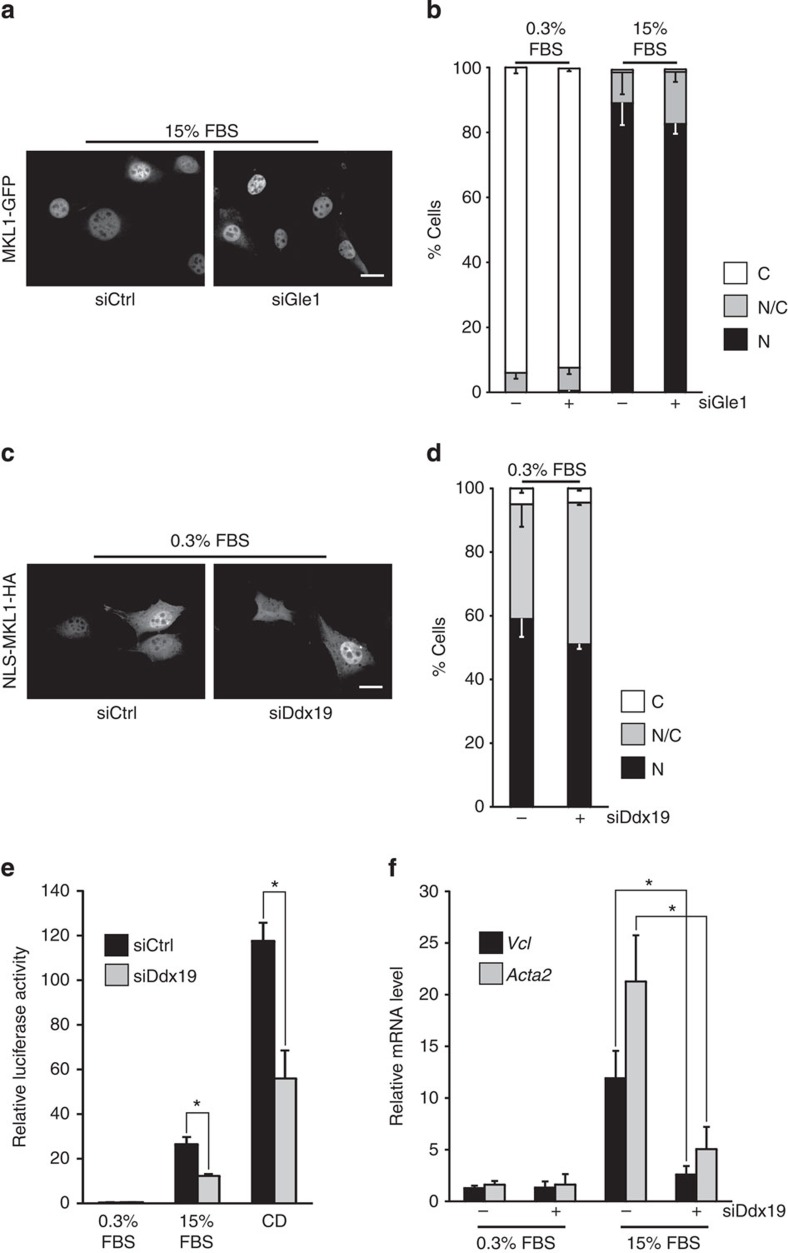
Ddx19 regulates the activity of the endogenous NLS in MKL1 and is required for SRF transcriptional activity. (**a**) Localization of MKL1-GFP in serum-stimulated (15% FBS) cells transfected with control or Gle1 siRNAs (si). Scale bar, 20 μm. (**b**) Quantification of MKL1 localization in control (−) or Gle1 (+) siRNA-transfected cells. C, cytoplasmic; N/C, pancellular; N, nuclear. One hundred cells per point; *n*=2; error bars are s.d. (**c**) Localization of NLS-MKL1-HA in serum-starved (0.3% FBS) cells transfected with control or Ddx19 siRNAs. Scale bar, 20 μm. (**d**) Quantification of NLS-MKL1 localization. One hundred cells per point; *n*=2; error bars are s.d. (**e**) SRF reporter activity in serum-starved (0.3% FBS), serum-stimulated (15% FBS) and cytochalasin D (CD)-treated NIH 3T3 cells transfected with control or Ddx19 siRNAs. *n*=3; error bars are s.e.m. *Statistically significant differences (*P*<0.05) tested by Student’s *t*-test. *P*-values: siCtrl versus siDdx19 (15% FBS) 0.0126; siCtrl versus siDdx19 (CD) 0.0147. (**f**) Expression of SRF target genes, *vinculin* (*Vcl*) and *Acta2*, in cells transfected with control (−) or Ddx19 (+) siRNAs and measured by quantitative reverse transcription–PCR using ΔΔCt method. Values were normalized to Gapdh. *n*=3; error bars are s.e.m. Statistics as in **e** with *P*-values: *Vcl* (transfected with control siRNA (−)) versus *Vcl* (transfected with Ddx19 siRNA (+)) 0.0333; *Acta2* (transfected with control siRNA (−)) versus *Acta2* (transfected with Ddx19 siRNA (−)) 0.0309.

MKL1 also displayed normal nuclear accumulation in cells depleted of Gle1 (Fig. 2a), which has an important role in mRNA export (Supplementary Fig. 1b,c) This demonstrates that a general block in mRNA export does not prevent nuclear accumulation of MKL1 and points to a more specific role for Ddx19 in nuclear import of MKL1.

To study if Ddx19 is regulating the activity of the endogenous NLS embedded within the RPEL domain of MKL1, or if it regulates the protein in some other way not directly related to the import process, we studied the localization of NLS-MKL1, which contains the SV40 NLS in the N terminus of MKL1 (ref. 5). Importantly, the additional NLS is targeted by the same import factors, Ipoα/β, as the endogenous NLS in MKL1 and this construct displays significant nuclear localization already in unstimulated cells (Fig. 2c,d). Remarkably, the nuclear localization of NLS-MKL1 was not sensitive to Ddx19 depletion (Fig. 2c,d), demonstrating that Ddx19 explicitly regulates the activity of the endogenous NLS in MKL1 and does not act, for example, by sequestering the protein in the cytoplasm.

The experiments above showed that Ddx19 is required for the nuclear localization of MKL1, which is a prerequisite for SRF activation through this pathway^7^. Indeed, depletion of Ddx19 considerably reduced SRF reporter activity on serum stimulation or Cytochalasin D treatment (Fig. 2e), which directly disrupts the MKL1-actin complex^5^. Moreover, Ddx19 depletion also reduced the serum-induced expression of endogenous MKL1-SRF target genes, *vinculin* and *Acta2* (Fig. 2f). These results underscore the importance of Ddx19 in SRF regulation through MKL1 nuclear localization.

### MKL1 import and mRNA export use distinct activities of Ddx19

**Figure 3:**
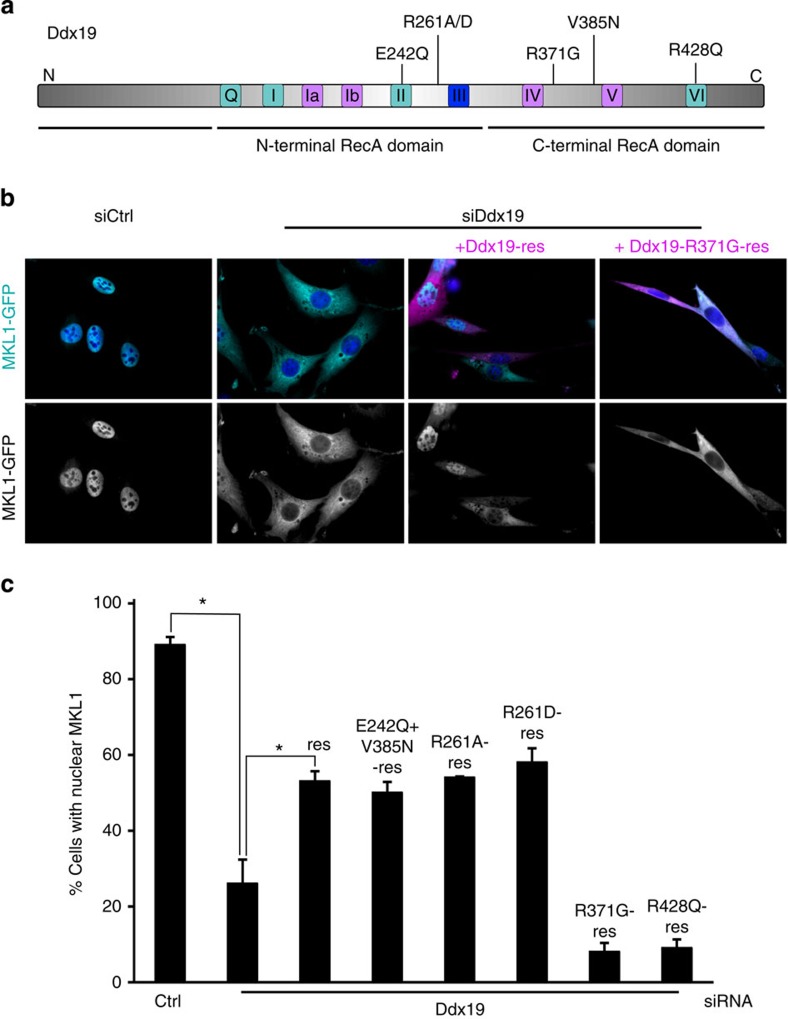
Helicase activity and Nup214 binding of Ddx19 are dispensable for MKL1 nuclear import but RNA binding is required. (**a**) Schematic presentation of Ddx19. Location of conserved sequence motifs that contribute to ATP binding/hydrolysis (coloured in cyan), RNA binding (magenta), or both (blue), within the Rec A helicase domains, and location of mutations used in **b**,**c** shown on top. (**b**) Localization of MKL1-GFP (cyan) and indicated Ddx19 constructs tagged with mCherry (magenta) in serum-stimulated cells. Nucleus is stained with DAPI (blue). Scale bar, 20 μm. (**c**) Quantification of MKL1 nuclear localization in Ddx19-depleted cells, which re-express the indicated Ddx19 point mutants. res, siRNA-resistant Ddx19; E242Q+V385N, double mutant that is defective for helicase activity; R261A and R261D, mutants that are defective for Nup214 binding; R371G and R428Q, mutants that are defective for RNA binding. One hundred cells per point; *n*=3; error bars are s.e.m. *Statistically significant differences (*P*<0.05) tested by Student’s *t*-test. *P*-values: Ctrl siRNA versus Ddx19 siRNA 0.00145; Ddx19 siRNA versus res (re-express siRNA-resistant Ddx19) 0.0226.

To study the biochemical requirements in Ddx19 needed for MKL1 import, we performed RNAi rescue experiments, where we reintroduced siRNA-resistant Ddx19 constructs (Fig. 3a and Supplementary Fig. 4) to depleted cells. Ddx19 helicase activity was disrupted by mutations in the conserved DEAD-box motif within the N-terminal RecA domain and in the carboxy-terminal RecA domain (Ddx19-E242Q,V385N; E240Q,V383N in yeast). This mutant has been shown to function in a dominant-negative manner in mRNA export, because it is defective for ATP binding and hydrolysis^16^. For sequence alignment of yeast Dbp5 and mouse Ddx19 proteins, see (Supplementary Fig. 3). To abrogate the interaction with Nup214, Ddx19 mutants R261A and R261D (R259 in yeast) were created^27^. This interaction has also been shown to be required for efficient mRNA export by triggering the ADP release from Ddx19 and enzyme recycling^19^. RNA binding of Ddx19 was disrupted by R371G (R369G in yeast) and R428Q (R426Q in yeast) mutations. Moreover, both of these mutants function as dominant negative in mRNA export^18^. We tested the functionality of the mutant constructs by measuring their ability to rescue the mRNA export defect in Ddx19-depleted cells (Supplementary Fig. 1b,c). As expected, none of the mutant constructs rescued the mRNA export phenotype (Supplementary Fig. 4a,b), supporting the notion that RNA binding, helicase activity and interaction with the nuclear pore are critical for Ddx19 to mediate mRNA export. Expression levels of the different mutant did not significantly vary (Supplementary Fig. 4c).

As mentioned earlier, both the MKL1 nuclear localization (Fig. 3b,c) and mRNA export phenotypes (Supplementary Fig. 4a,b) were rescued by expressing siRNA-resistant wild-type Ddx19. Surprisingly, the helicase-defective mutant (Ddx19-E242Q,V385N) was able to rescue the MKL1 localization to a similar extent as the wild-type Ddx19 (Fig. 3c), suggesting that the helicase activity of Ddx19 is not required for nuclear import of MKL1. Similarly, the two mutants unable to bind Nup214 (Ddx19-R261A and Ddx19-R261D) rescued the MKL1 nuclear import (Fig. 3c). This suggests that Ddx19 operates in the cytoplasm, and not on the nuclear pore, to regulate MKL1 localization. Interestingly, the only constructs that failed to rescue MKL1 localization were the Ddx19-R371G and Ddx19-R428Q mutants, which are defective in RNA binding (Fig. 3b,c). In fact, both mutants act in a dominant-negative manner, because their expression caused nuclear exclusion of MKL1 in serum-stimulated conditions even in control siRNA-treated cells (Supplementary Fig. 5a,b).

### Ddx19 interacts with the RPEL domain of MKL1

**Figure 4:**
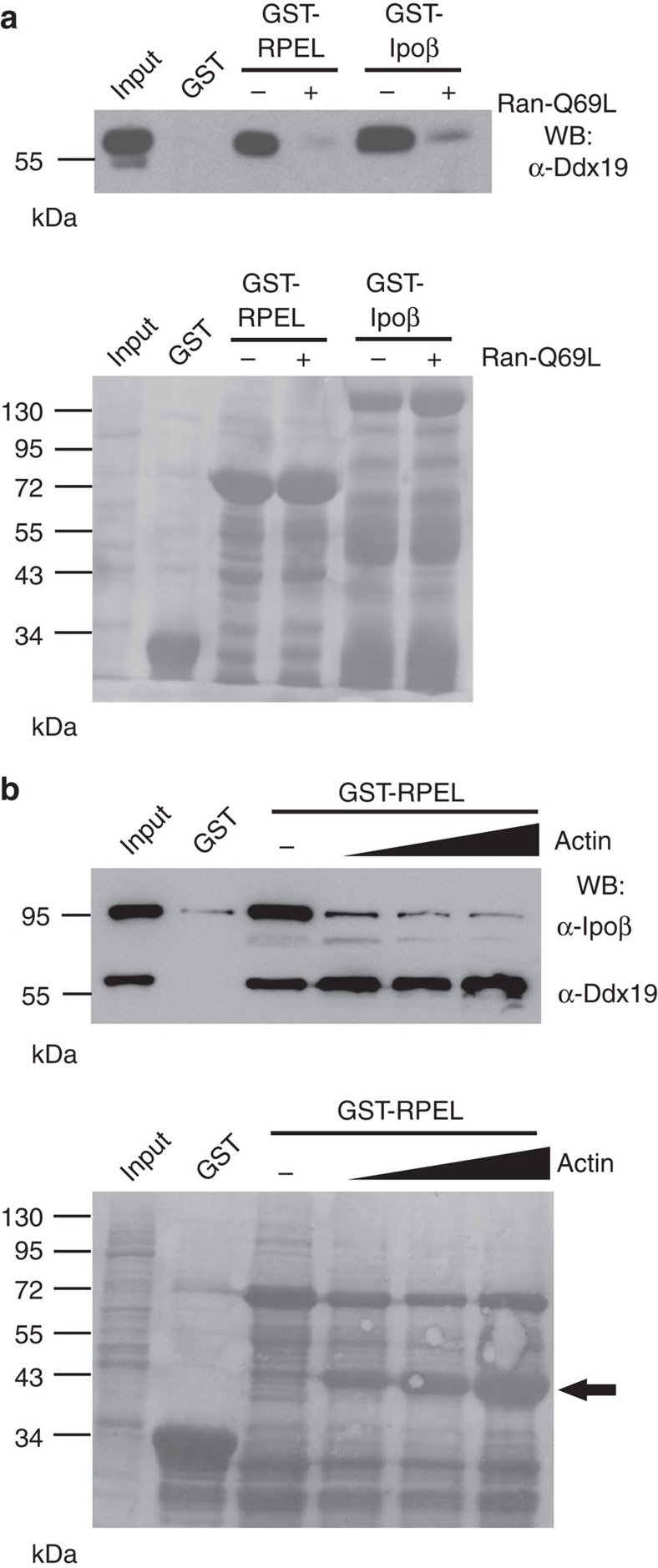
Ddx19 interacts with MKL1 RPEL domain and Ipoβ. (**a**) Ddx19 recruitment from Hela cell lysate by GST-RPEL and GST-Ipoβ (left). WB, western blotting; (−), without Ran-Q69L; (+), with RanQ69L. Corresponding GST baits are stained with Ponceau to ensure the equal loading of the samples (below). Input sample corresponds to 5% of the Hela cell lysate used in the assay. (**b**) GST-RPEL was used as a bait for pull down of Ddx19 and Ipoβ from the Hela cell lysate in the presence of increasing amounts of LatB-actin (0.25–10 μM) (left). Arrow indicates the increasing amounts of actin in the corresponding Ponceau staining (below).

The RPEL domain of MKL1 has been shown to be both necessary and sufficient for MKL1 nucleo-cytoplasmic shuttling^11^ and the Ipoα/β-heterodimer binds to this domain in an actin-dependent manner^6^. The fact that Ddx19 seemed to regulate the nuclear localization of the endogenous NLS of MKL1 located within the RPEL domain (Fig. 2d) prompted us to investigate whether Ddx19 would also interact with this part of the MKL1 protein. To study the interactions between MKL1-RPEL domain, Ipoβ and Ddx19, we performed a glutathione *S*-transferase (GST) pull-down assay from cytosolic Hela cell lysate. In this assay, Ddx19 interacted with both GST-MKL1-RPEL and GST-Ipoβ (Fig. 4a). Interestingly, both interactions were disrupted by the addition of Ran-Q69L mutant (Fig. 4a), which permanently locks Ran in its GTP-bound state^28^, and thus mimics nuclear conditions. The interaction between Ddx19 and MKL1/Ipoβ therefore resembles a classical nuclear import cargo complex.

**Figure 5:**
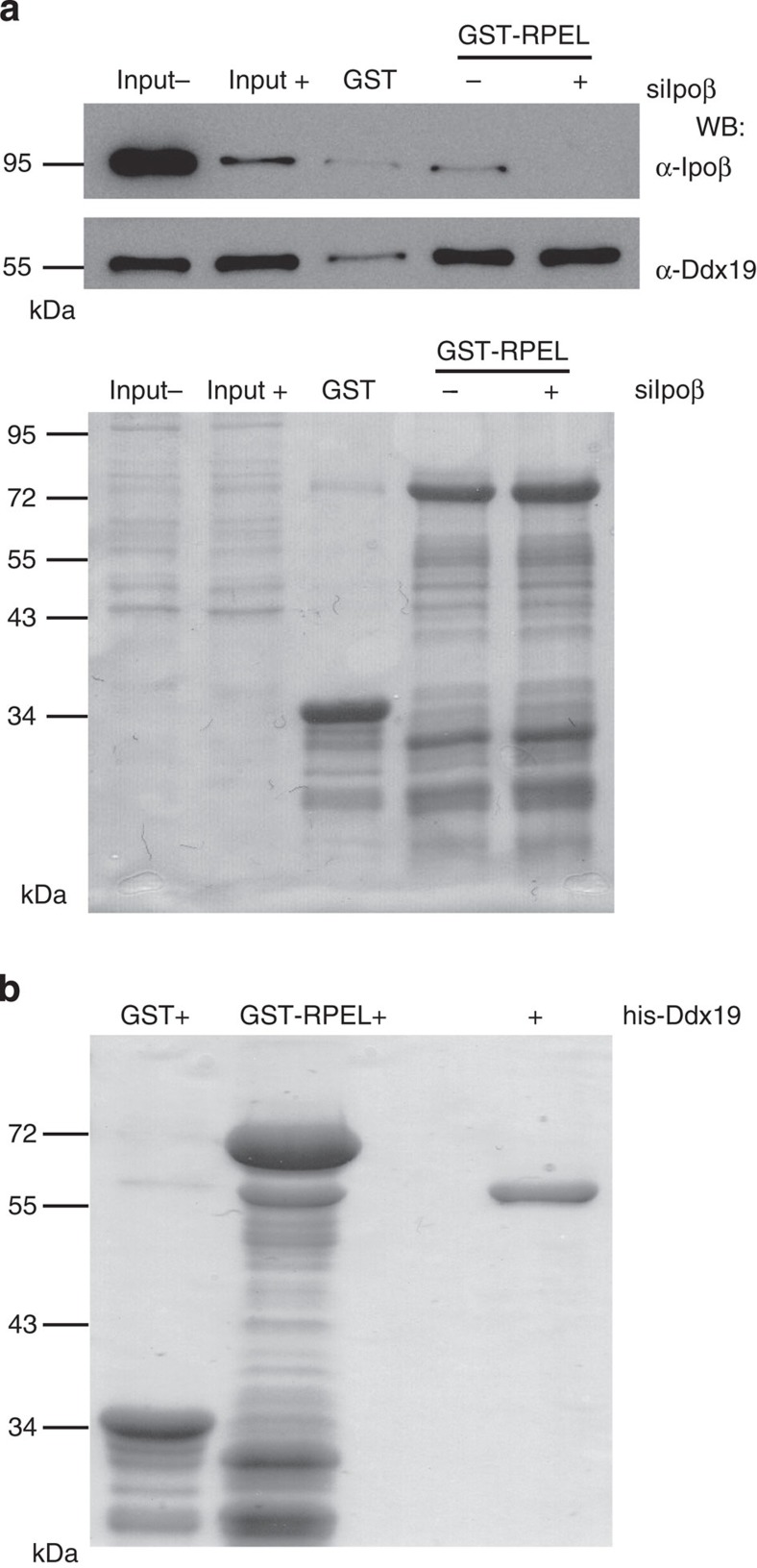
Ddx19 binds directly to MKL1 RPEL domain (**a**) Recruitment of Ipoβ and Ddx19 from Ipoβ-depleted NIH 3T3 cytoplasmic lysates by GST-RPEL. (−), NIH 3T3 cytoplasmic lysate transfected with control siRNAs; (+), Ipoβ-depleted NIH 3T3 cytoplasmic lysate. Ponceau-stained full membrane (below). (**b**) Purified his-tagged Ddx19 (+; 54 kDa right lane) binds directly to GST-RPEL (middle lane) but not to GST alone (left lane). Proteins are stained with Coomassie brilliant blue.

Actin regulates MKL1 localization and its overexpression inhibits MKL1 nuclear import *in vivo*^5^,^7^ and its interaction with the RPEL domain *in vitro*^6^. To gain insights into whether actin also affects Ddx19 binding, we titrated nonpolymerizable Latrunculin B (LatB)-actin into the pull-down assay. As described before^6^, when the actin concentration was increased, the Ipoβ binding was correspondingly decreased. However, no effect on Ddx19 binding was observed (Fig. 4b). Thus, actin binding does not compete with Ddx19 binding for the MKL1 RPEL domain as it does with the Ipoα/β complex. This data also suggested that binding of Ddx19 to MKL1 RPEL domain is not dependent on Ipoβ. To test this directly, we prepared cytoplasmic lysates of cells depleted of Ipoβ for the GST pull-down assay. As expected, based on the actin titration experiment (Fig. 4b) Ddx19 bound as efficiently to GST-MKL1-RPEL from the Ipoβ−depleted extract as from the control extract (Fig. 5a). Importantly, the interaction between MKL1-RPEL and Ddx19 is direct, because purified Ddx19 expressed in a baculoviral system efficiently bound to recombinant MKL1-RPEL domain (Fig. 5b). Previous studies with purified recombinant proteins have shown that the MKL1-RPEL domain can interact directly with Ipoα/β^6^. Accordingly, Ddx19 depletion did not affect binding of Ipoβ to MKL1-RPEL domain (Supplementary Fig. 6). This demonstrates that the nuclear import defect of MKL1 in the Ddx19-depleted cells cannot be explained by reduced Ipoβ-binding to the isolated RPEL domain of MKL1.

**Figure 6:**
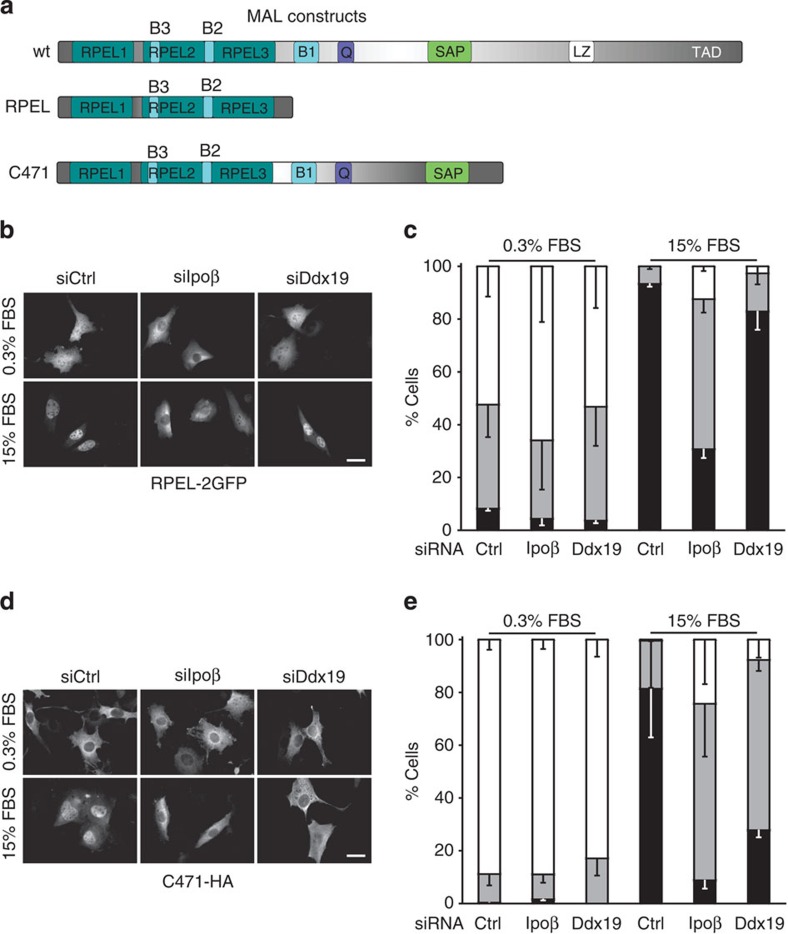
Ddx19 influences the localization of longer MKL1-C471 construct but not the RPEL domain alone. (**a**) Schematic presentation of MKL1 constructs used in the study. (**b**) Localization of MKL1-RPEL domain in serum-starved (0.3% FBS) and -stimulated (15% FBS) cells transfected with control, Ipoβ or Ddx19 siRNAs (si). Scale bar, 20 μm. (**c**) Quantification of MKL1-RPEL localization. C, cytoplasmic; N/C, pancellular; N, nuclear. One hundred cells per point; *n*=2; error bars are s.d. (**d**) Localization of MKL1-C471 construct in cells transfected with negative control, Ipoβ or Ddx19 siRNAs (si). (**e**) Quantification of MKL1-C471 localization in NIH 3T3 cells. One hundred cells per point; *n*=2; error bars are s.d.

Our results so far suggest that Ddx19 might add a second level to the classical actin-based regulation of MKL1 localization. As mentioned, the RPEL domain of MKL1 displays very similar nucleo-cytoplasmic shuttling behaviour compared with the full-length protein^11^. As all the previous assays had examined the localization of full-length MKL1 (Figs 1b,c, 2c,d and 3a,b), we next studied the localization of MKL1-RPEL-2GFP^11^ (Fig. 6a) in Ipoβ- and Ddx19-depleted cells. Depletion of Ipoβ efficiently prevented the nuclear accumulation the MKL1-RPEL domain as described earlier^6^, but interestingly, depletion of Ddx19 did not have any effect on the localization of this construct (Fig. 6b,c). Therefore, despite interacting with the RPEL domain, Ddx19 is dispensable for its subcellular localization in intact cells. This suggests that Ddx19 engages also other regions within MKL1.

To study this further, we next decided to test the localization of a longer MKL1 construct, MKL1-C471, which, in addition to the RPEL domain, also includes B1, Q and SAP regions (Fig. 6a). The MKL1-C471 behaved very similarly to the full-length MKL1 (compare quantification in Fig. 1c) and its nuclear accumulation was clearly reduced in cells depleted either of Ipoβ or Ddx19 (Fig. 6d,e).

**Figure 7:**
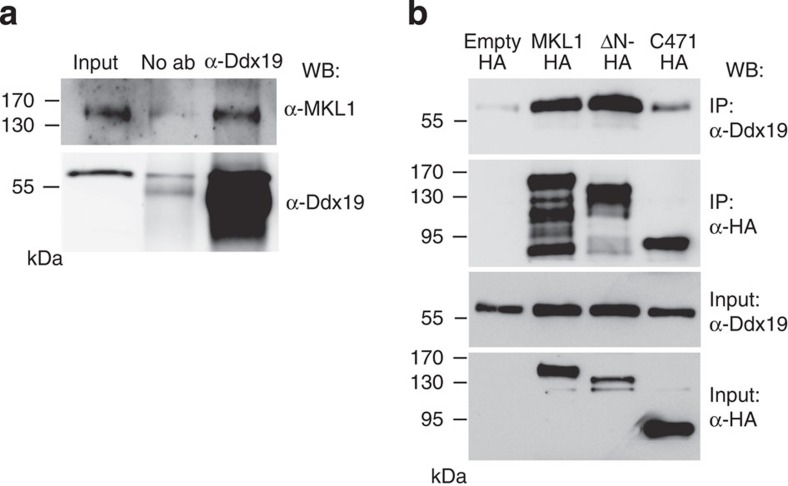
MKL1 contains two Ddx19 interaction sites. (**a**) Co-IPs of endogenous MKL1 with endogenous Ddx19. NIH 3T3 cytoplasmic extract was immunoprecipitated with anti-Ddx19 antibody and the immunoprecipitates were blotted for indicated antibodies. Beads without antibody (no ab) were used as a negative control. (**b**) HA-tagged MKL1 constructs were immunoprecipitated from NIH 3T3 lysates and their expression was detected by HA-antibody. Co-precipitated endogenous Ddx19 was detected by western blotting (WB). Empty HA was used as a negative control.

To study the interaction between full-length MKL1 and Ddx19, we performed co-immunoprecipitations (co-IP). Ddx19 antibodies efficiently precipitated endogenous MKL1 from cell lysates (Fig. 7a), demonstrating that the two proteins also interact in the cellular context. Our localization studies with the different-length MKL1 constructs (Fig. 6) suggested that in addition to the RPEL domain, Ddx19 might also engage more C-terminal sequences of MKL1. Indeed, MKL1-ΔN, which lacks the N terminus including the RPEL domain efficiently co-precipitated Ddx19 from cell lysates (Fig. 7b). This demonstrates that MKL1 contains two Ddx19 interaction sites: the direct binding site in the RPEL domain (Fig. 5b) and a second site, direct or indirect, in the C terminus. Moreover, the MKL1-C471 construct, which is dependent on Ddx19 for its nuclear localization (Fig. 6d,e), bound to Ddx19 (Fig. 7b), albeit less than the full-length MKL1 and MKL1-ΔN.

### Ddx19 regulates MKL1 conformation for efficient Ipoβ binding

Although Ddx19 was dispensable for Ipoβ binding to MKL1 RPEL domain in an *in vitro* assay, we decided to study the interaction between the full-length MKL1 and Ipoβ in living cells by using Förster Resonance Energy Transfer (FRET) measured by Fluorescence Lifetime Imaging (FLIM). MKL1-GFP was used as the donor and Ipoβ-mCherry was used as the acceptor. Soluble green fluorescent protein (GFP) and GFP-mCherry tandem construct were used to calibrate the system (Supplementary Fig. 7a). The fluorescence lifetime of MKL1-GFP was significantly reduced from 2.50 to 2.41 ns in the presence of Ipoβ-mCherry construct (Fig. 7a), indicating a close proximity of GFP and mCherry tags, and thus an interaction between MKL1 and Ipoβ, as demonstrated previously in a proximity ligation assay^6^. Significantly, in Ddx19-depleted cells, the fluorescence lifetime of MKL1-GFP was the same in the presence and absence of the FRET acceptor Ipoβ-mCherry (Fig. 7a). Ddx19 is thus required for efficient interaction between MKL1 and its nuclear import receptor, Ipoβ, *in vivo*. Of note, expression of soluble mCherry did not affect MKL1-GFP lifetimes (Supplementary Fig. 7b), demonstrating the specificity of the assay. Importantly, in the presence of Ipoβ-mCherry, the MKL1-RPEL-GFP displayed a similar decrease in its fluorescence lifetime in both control and Ddx19-depleted cells (Fig. 7a), signifying that Ddx19 does not play a role in Ipoβ interactions with the RPEL domain. This confirms our *in vitro*-binding assays (Supplementary Fig. 6) and localization studies (Fig. 6b,c) using this construct. Our results therefore suggest that some C-terminal sequences may have an inhibitory effect on MKL1 import, which led us to speculate that perhaps Ddx19 is involved in controlling the conformation of the full-length MKL1 protein.

**Figure 8:**
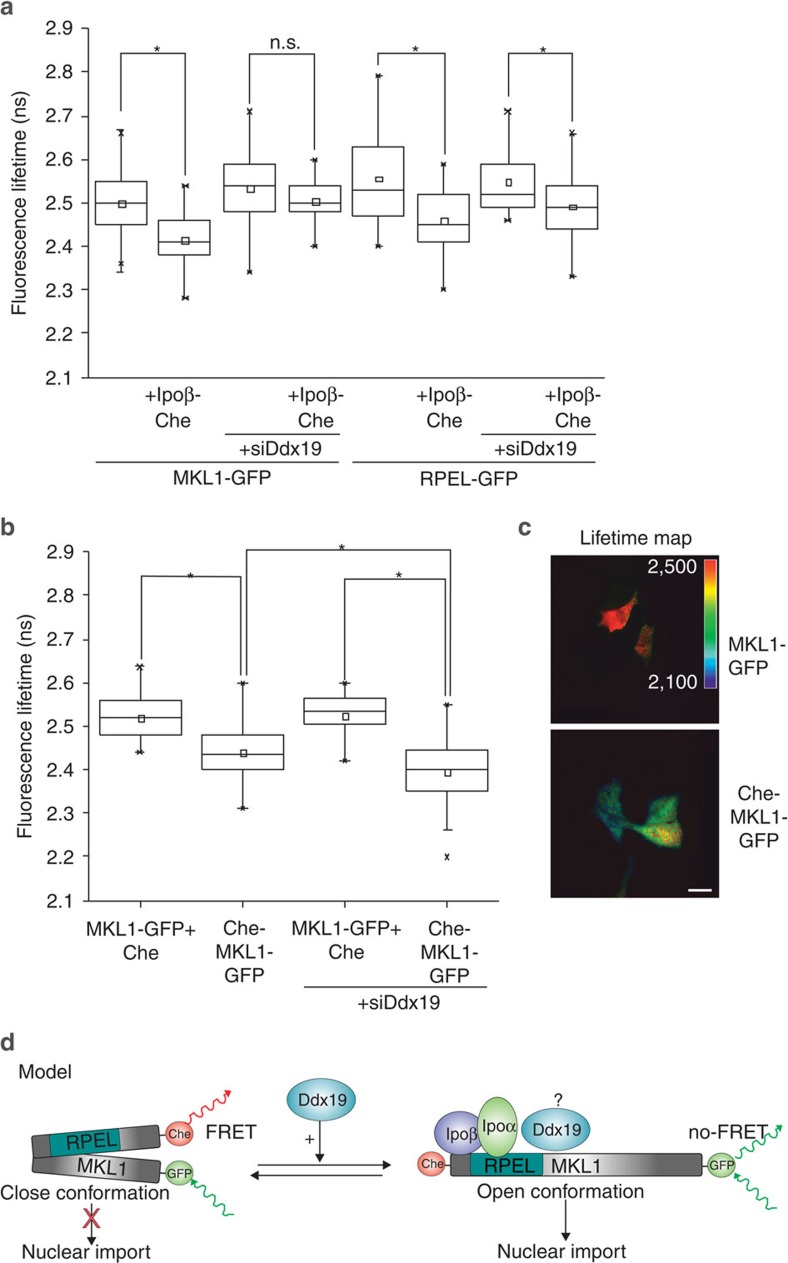
Ddx19 helps to maintain an open configuration of MKL1, which is required for Ipo binding. (**a**) Detection of MKL1-Ipoβ and RPEL-Ipoβ interaction by FRET/FLIM from control or Ddx19 siRNA-treated cells. Fluorescence lifetimes of MKL1-GFP constructs are shown as box-and-whisker plots, where the upper and lower limits of the box correspond to the 75th and 25th percentiles, the median is indicated by the central bar and the whiskers indicate the minimum and maximum values. Twenty one to 111 cells per condition. *Statistically significant differences tested by Mann–Whitney test with 0.05 significance level. n.s., not significant. *U*-values from left to right: 7.49178E−13, 0.20604, 4,8184E−5 and 0.03118. (**b**) FRET/FLIM between mCherry and GFP was used to study MKL1 conformation. Fluorescence lifetime of the C-terminal GFP donor (MKL1-GFP) co-transfected with free mCherry (Che) was compared with that of the same donor in a construct containing an N-terminal mCherry (Che-MKL1-GFP) acceptor. Fluorescence lifetimes are shown as box-and-whisker plots as above. Twenty one to 111 cells per condition. Statistics as in a, with *U*-values: MKL1-GFP+Che versus Che-MKL1-GFP 4.33286E−7; Che-MAL-GFP versus siDdx19 Che-MKL1-GFP 0.01771; siDdx19 MKL1-GFP+ Che versus siDdx19 Che-MKL1-GFP 1.96729E−8. (**c**) Lifetime maps of the MKL1-GFP and Che-MKL1-GFP. Scale bar, 20 μm. (**d**) A schematic model of how Ddx19 may regulate MKL1 nuclear import. In the absence of Ddx19, MKL1 N and C terminus are spatially close to each other, allowing FRET between N- and C-terminal fused mCherry and GFP. This closed conformation does not permit Ipoβ binding and thus is not compatible with MKL1 nuclear import. Ddx19 facilitates the acquisition of an open conformation that allows the proper recruitment of Ipoα/β for efficient MKL1 nuclear import. In the open conformation, the GFP and mCherry fusions are further apart and no FRET occurs. Whether Ddx19 travels together with MKL1-Ipoβ to the nucleus remains to be determined and this is indicated with a question mark.

To directly test this, we created a FRET-based reporter to study conformational changes in MKL1 by fusing mCherry and GFP tags to the N- and C-terminal ends of the protein, respectively (Fig. 8d). We then measured the FRET between GFP and mCherry by FLIM, and noted a small but significant decrease in the fluorescence lifetime (indicative of FRET between enhanced GFP (EGFP) and mCherry) in the mCherry-MKL1-GFP construct (2.44 ns) compared with the fluorescence lifetime of MKL1-GFP with free mCherry (2.52 ns) (Fig. 8b,c). This indicates that in the context of the full-length protein, the N and C terminus of MKL1 come close to each other (<10 nm) to permit energy transfer between the fused fluorescent proteins, GFP and mCherry. Significantly, the fluorescence lifetime of the mCherry-MKL1-GFP construct decreased even further to 2.40 ns in Ddx19-depleted cells (Fig. 8b). Therefore, the absence of Ddx19 seems to promote a closed conformation of MKL1. These results strongly support a model in which Ddx19 regulates MKL1 conformation and helps to maintain an open configuration that can be recognized by Ipoα/Ipoβ for efficient nuclear import (Fig. 8d).

## Discussion

Regulation of gene expression is one of the most fundamental aspects of the whole cell. It is a multistep process, which entails flow of information and macromolecules between the two main compartments of the cell, the cytoplasm and the nucleus. Coordination of these events is crucial for precise gene expression and thus elemental for every cell. We have here uncovered a novel role for Ddx19, an established mRNA export factor and translation regulator, in nuclear import of a key transcriptional regulator, MKL1. We thus propose that Ddx19 plays an important role in coordinating the key nucleo-cytoplasmic shuttling events in gene expression by linking transcriptional activation, mRNA export and translation.

Controlled nuclear import and export are at the heart of MKL1 regulation. In unstimulated cells, MKL1 localizes to the cytoplasm, but it accumulates rapidly in the nucleus to activate SRF on stimulations that polymerize actin^5^,^7^. We have shown before that nuclear import of MKL1 is dependent on the classical nuclear import pathway mediated by the Ipoα/β complex. The NLS is embedded in the N-terminal RPEL domain of MKL1, and structural and biochemical studies have shown that actin and the Ipoα/β-complex compete for binding MKL1 (refs 6, 13). Here we add an additional layer of regulation, which is not dependent on actin, by demonstrating the involvement of Ddx19 in nuclear import of MKL1 (Fig. 1b,c). Many of our experiments show that this effect is specific, and that Ddx19 is not a general nuclear import factor. First, all of our control proteins localized in the nucleus in Ddx19-depleted cells (Fig. 1d,e). Among these, the NLS-2GFP-NES construct uses exactly the same transport factors for nuclear import and export, Ipoα/β complex and Crm1, respectively, as MKL1 does. This signifies that Ddx19 depletion does not operate by blocking the function of these key nuclear transport mediators. The effect of Ddx19 was also not an indirect consequence of mRNA nuclear accumulation, because depletion of Gle1, which also causes the same mRNA retention phenotype (Supplementary Fig. 1b,c), did not affect MKL1 nuclear localization (Fig. 2a,b). Moreover, expression of Ddx19 mutants (see below) that have a dominant-negative role in mRNA export^15^,^16^ did not block MKL1 import (Supplementary Fig. 5b), but efficiently rescued the RNAi phenotype of Ddx19 (Fig. 3c). As Gle1-Ddx19 interaction is also required for translation^22^,^29^, it is also not likely to be that the nuclear import defect is due to unspecific effects on protein synthesis. This is also further supported by the notion that the helicase activity of Ddx19 is required for translation termination^22^, but not for MKL1 import (Fig. 3c). In the future, it will be interesting to study whether Ddx19 could regulate the nuclear import of other transcription factors as well.

To facilitate nuclear export, Ddx19 interacts with mRNA, Gle1-IP_6_ and the cytoplasmic fibrils of the NPCs via Nup214 and uses it’s ATPase cycle to remodel and release the mRNP to the cytoplasm^17^. To understand which biochemical activity of Ddx19 is required for nuclear import of MKL1, we used a set of mutants, where these key activities are abolished by point mutations (Fig. 3a and Supplementary Fig. 3). Importantly, we also tested the functionality of the mutants in mRNA export and, as expected and reported previously, found that all of the above-mentioned activities were required for this process (Supplementary Fig. 4a,b). However, the ATPase/helicase activity or Nup214 binding by Ddx19 were dispensable for MKL1 nuclear import (Fig. 3c), supporting the idea that the mRNA export/translation functions are separate from MKL1 import. Moreover, as the association with the NPC was not needed, Ddx19 operates in the cytoplasm to promote MKL1 import. Concordantly, Ran-GTP, which is abundant in the nucleus, abolishes the interaction between MKL1 and Ddx19 (Fig. 4a). In cells, Ddx19 localizes both to the nuclear envelope as well as to the cytoplasm^15^,^16^. The functional relevancy of cytoplasmic Ddx19 was for a long time unclear, but then the role in translation termination was proposed^22^, and now our results uncover the second cytoplasmic function for Ddx19 in nuclear import of a specific transcriptional regulator. Of note, Ddx19 also shuttles in and out of the nucleus, because LMB treatment, which inactivates the export receptor Crm1, accumulates it into the nucleus^15^. This indicates that Ddx19 may also have a nuclear role and, indeed, Dbp5 has been localized to transcriptionally active gene loci in the *Chironomus tentans* salivary glands^30^ and *Saccharomyces cerevisiae* Ddp5 interacts with components of the TFIIH complex^31^. Although unlikely to play a role in MKL1 regulation, the nuclear pool of Ddx19 may further expand the roles of this protein in controlling multiple steps of the gene expression process.

Interestingly, although the helicase activity of Ddx19 is not needed for MKL1 import, the RNA-binding capacity is (Fig. 3b,c and Supplementary Fig. 5a,b). This was a slightly unexpected finding, because the interaction between mRNA and Ddx19 for nuclear export should take place at the NPC, and our results demonstrate that Ddx19 operates in the cytoplasm to regulate MKL1 localization (Fig. 3). Our results therefore hint at the possibility that Ddx19 might bind and regulate mRNAs also in the cytoplasm, which is not surprising considering its role in translation. Although we cannot completely rule out the possibility that the used mutants, Ddx19-R371G and Ddx19-R428Q, that we used to abolish RNA binding^18^ also have other uncharacterized consequences on Ddx19 function, it is tempting to speculate that MKL1 localization responds to a specific subset of Ddx19–mRNA complexes. So far, no sequence specificity has been described for Ddx19 mRNA binding. This aspect, as well as the possible selective interaction between Ddx19 and mRNAs in the different compartments of the cell (nucleus, NPC and cytoplasm), warrants further investigation.

MKL1 RPEL domain has been shown to be both necessary and sufficient for MKL1 localization^11^, because it includes all the elements for actin-dependent nucleo-cytoplasmic shuttling^6^,^12^. However, also regions outside the RPEL domain have been suggested to play a role in nuclear localization of MKL1. For example, the B1 region, which also mediates the interactions between MKL1 and SRF, is required for nuclear accumulation of at least the shorter MAL(met) construct and MKL1 derivatives lacking the N-terminal sequences^7^,^32^, while the Q domain has been implicated in Crm1 binding^12^. In our hands, the MKL1-RPEL domain always displayed slightly more diffuse localization compared with the full-length protein (Figs 1c and 6b,c), hinting that sequences outside the RPEL domain could contribute to full regulation of MKL1 subcellular localization. To our initial surprise, we found that Ddx19 was dispensable for nuclear localization of MKL1 RPEL domain (Fig. 6b,c), although it efficiently and directly bound to it (Figs 4a and 5b). Ddx19 is therefore the first protein to be characterized that influences the localization of only longer MKL1 constructs. We first speculated that Ddx19 might regulate dimerization of MKL1, which has been suggested to play a role in nuclear export of MRTF family members^12^. However, Ddx19 was required for nuclear import of MKL1-C471 construct^7^, which lacks the leucine zipper region mediating the homo- and heterodimerization between the MRTF family members^12^,^33^. This lead us to speculate that Ddx19 could be involved in regulating the conformation of MKL1, by interacting with both the RPEL domain (Figs 4a,b and 5a,b) and some more C-terminal sequences that were also detected in our co-IP experiments (Fig. 7b). Our FRET assays performed in live cells proved this directly (Fig. 8b,c). MKL1 is a relatively large protein of ~100 kDa and the only structural information so far is from the N-terminal RPEL domain, which constitutes only ~25% of the protein. Despite the fact that defined regions within the C terminus of MKL1 have been assigned with specific functions, such as the SRF-binding B1 box and the C-terminal transcription activation domain^7^,^34^, there is no data on the three-dimensional arrangement of the full-length protein. Our FRET assay demonstrates that *in vivo*, the N and C termini of MKL1 come very close to each other, and that in the absence of Ddx19 this interaction occurs more readily (Fig. 8b,c).

Our data therefore points to a model, where Ddx19 impinges on MKL1 import by facilitating the acquisition of an open conformation, which can then efficiently interact with Ipoβ for nuclear import (Fig. 8d). Indeed, depletion of Ddx19 decreased the interaction between full-length MKL1 and Ipoβ in intact living cells in our FRET assay, but did not have a significant effect on MKL1-RPEL domain–Ipoβ interaction (Fig. 8a). The latter experiment fully agrees with our *in vitro* GST pull-down experiments (Fig. 5b). The switching between the ‘open’ and ‘closed’ MKL1 conformations is likely to be very dynamic. In serum-starved cells, MKL1 is known to constantly and rapidly shuttle between the nucleus and cytoplasm^5^, and therefore the import-incompetent closed conformation must be readily converted to the import-competent open conformation. Detailed molecular understanding of how Ddx19-Ipoβ-MKL1 interactions determine the nuclear import status of MKL1 demand structural information from the C-terminal parts of MKL1 and mapping of the exact Ddx19-binding site. Whether the B1 region, previously implicated in nuclear import of some MKL1 constructs^32^, plays a role here warrants further investigation, although our preliminary analysis indicates that MKL1-ΔB1-GFP construct is also dependent of Ddx19 for its nuclear localization. Interestingly, conformation changes have also previously been suggested to play a role in MKL1 localization, as phosphorylation of serine 454 in the middle parts of the protein seems to affect both actin-binding and nuclear accumulation of MKL1 (ref. 35).

As Ddx19-MKL1 interaction is not regulated by actin (Fig. 4b), our data also suggest that actin dynamics may not be the sole determinant of MKL1 subcellular localization in cells. The relationship and possible regulatory interactions between nuclear transport of mRNA and MKL1 remain to be discovered. As the same protein, Ddx19, is involved in both processes, it is nevertheless tempting to speculate that in addition to responding to actin dynamics, the MKL1-SRF pathway could also monitor the general gene expression landscape of the cell through Ddx19–mRNA interaction. This idea is supported by the notion that RNA binding seems to be critical for Ddx19 to regulate MKL1 nuclear localization. Perhaps decreased Ddx19–mRNA interaction, due to, for example, inhibited transcription or pre-mRNA processing in stress conditions, will inhibit the MKL1-SRF transcriptional response, and thus dampen actin dynamics to save energy. In the future, it will also be interesting to assess how the role of Ddx19 in translation termination fits to this scheme and whether MKL1 is the only transcription factor regulated by this DEAD-box helicase. Our studies have therefore broadened the role of Ddx19 in gene expression from regulating the fate of mRNAs to controlling transcription factor activity.

## Methods

### Plasmids and antibodies

Details of the plasmids cloned for the study are available on request.

Plasmids that have been previously described include: NLS-MKL1 (ref. 5); MKL1(2-263)-2GFP-N3 (RPEL), MC-pEGFP-C1 and pET41a-3CΔ-MKL1(2-261)^11^; MKL1(fl)-C471 (ref. 7); and SRF-VP16 (ref. 36). RanBPI-ΔNES-pEGFP-C1 and Smad4-pEGFP-C1 were kind gifts from Caroline Hill, pQE-RanQ69L from Maarten Fornerod and pRL-TK was from Promega.

The following antibodies were from Sigma: Flag (M2, F1804, 1:300), haemagglutinin (HA-7, G21234, 1:300), Ipoβ (31H4, I2534, 1:2,000) and Tubulin (B512, T6074, 1:2,000). MKL1 (C-19, SC-21558, 1:100) antibody was from Santa Cruz Biotechnology and Ddx19 (NB100-760, 1:2,000) was from Novus Biologicals. The following secondary antibodies were from Invitrogen: horseradish peroxidase (HRP)-conjugated anti-mouse (G21040, 1:5,000), HRP-conjugated anti-rabbit (G21234, 1:5,000), HRP-conjugated anti-goat (A9452, 1:5,000), Alexa-Fluor-488-conjugated anti-mouse (A21155, 1:500), Alexa-Fluor-488-conjugated anti-rabbit (A11008, 1:500), Alexa-Fluor-594-conjugated anti-mouse (A11005, 1:500), Alexa-Fluor-594-conjugated anti-rabbit (A11012, 1:500) and Alexa-Fluor-594-conjugated anti-goat (A11080, 1:500).

### Cell culture with immunofluorescence and microscopy

Mouse embryonic fibroblast cell line (NIH 3T3) was cultured in DMEM (Lonza) containing 10% fetal bovine serum (FBS; GIBCO), 100 Units ml^−1^ Penicillin and 0.1 mg ml^−1^ Streptomycin (Pen-Strep, GIBCO). Tetracycline-inducible MKL1-GFP-expressing mouse embryonic fibroblast (R332)^5^ and human breast adenocarcinoma MCF7 (M10A12) cell lines were cultured in DMEM containing 10% FBS, Pen-Strep, 5 μg ml^−1^ Blasticidin (Invivogen) and 250 μg ml^−1^ Zeocin (Invivogen). All cell lines were obtained from the lab of Richard Treisman and were grown in humidified 95% air/5% CO_2_ incubator at +37 °C.

For transfections, NIH3T3, R332 or M10A12 cells were plated onto 24-well tissue culture plate at a density of 12,000 cells per well. The next day, cells were transfected with DNA constructs using JetPrime transfection reagent (Polyplus transfection), according to the manufacturer’s instructions. In total, 200 ng of DNA was used for every transfection reaction, with the experimental vector amount varying from 25 to 50 ng depending on the application. The pEF-vector was used to fill up the total amount of DNA. After 5 h, the media was changed to fresh growth media containing 0.3% FBS and the following day treated with FBS (15%), LMB (20 nm, Calbiochem) or Cytochalasin D (2 μM, Sigma) for desired time point where indicated, before processing the samples for western blotting, microscopy or luciferase assay. When using R332 or M10A12 cells, tetracycline (1 μg ml^−1^) was added to the growth medium after transfection to induce the expression of MKL1-GFP.

For siRNA transfections, NIH3T3 cells or R332 cells were plated onto 24-well tissue culture plate at a density of 5,000 cells per well. The following day, cells were transfected with 10 nmol siRNA (mouse Ddx19ab: 5′-GAAGCUAGCUUAUGCUGUU-3′ and 5′-AACAGCAUAAGCUAGCUUC-3′ from Qiagen; human Ddx19ab: 5′-UCAACAAGCUGAUCAGAAG-3′ and 5′-CUUCUGAUCAGCUUGUUGA-3′ from Sigma; negative control: AllStars negative control from Qiagen; mouse Ipoβ: 5′-CAACUGAAACCAUUAGUCA-3′ and 5′-UGACUAAUGGUUUCAGUUG-3′ from Sigma; mouse Gle1: 5′-CCUAAAGCUUCGAGAGGCA-3′ and 5′-UGCCUCUCGAAGCUUUAGG-3′ from Sigma) using Interferin transfection reagent (Polyplus transfection), according to manufacturer’s instructions. On day 4, cells were re-transfected with siRNAs and with DNA constructs, if needed (see above), and the following day processed for western blotting, microscopy or luciferase assay. When using R332 or M10A12 cells, tetracycline (1 μg ml^−1^) was added to the growth medium, to induce the expression of MKL1-GFP.

Fluorescence *in situ* hybridization experiments were done as described earlier in the protocol developed in the lab of Robert Singer ( http://www.singerlab.org/protocols). Briefly, cells in a 24-well plate were transfected with siRNAs as above. On the fourth day, cells were transfected, if needed, with Ddx19-constructs using JetPrime transfection reagent and the following day fixed with 4% formaldehyde, 10% acetic acid in 1 × PBS (10 mM Na_2_HPO_4_, 1.5 mM KH_2_PO_4_ pH 7.4, 140 mM NaCl, 3 mM KCl). Cells were permeabilized by treatment with 70% ethanol o/n at +4 °C and the following day rehydrated with wash buffer (15% Formamide (Ambion), 2 × SSC (Ambion), nuclease-free water) for 5 min at room temperature. The hybridization was done at +37 °C for 3 h using 10 ng of fluorescently labelled Alexa 488-PolydT probe (Sigma) diluted in hybridization buffer (10% Dextran sulfate (Sigma), 10 mg *Echerichia coli* transfer RNA (Roche), 2 mM Vanadyl ribonucleoside complex (New England Biolabs), 0.02% RNAse-free BSA (New England Biolabs), 2 × SSC, 15% formamide and nuclease-free water). After two washes, cells were processed for microscopy.

For microscopy, cells were fixed with 4% paraformaldehyde for 10 min, washed three times with PBS and permeabilized for 5 min with 0,2% Triton X-100 in PBS. For antibody staining, permeabilized cells were blocked with blocking buffer (1% BSA, 1% Gelatin, 10% FBS) for 30 min and incubated with primary antibody for 1 h. Coverslips were washed and incubated with Alexa Fluor-conjugated secondary antibody for 1 h with or without DAPI (4',6-diamidino-2-phenylindole). Coverslips were washed three times with PBS and mounted in moviol supplemented with DABCO. Cells were imaged using × 63 objective of Axio Imager M2 equipped with AxioCam HRm camera and AxioVision software (Zeiss).

### Luciferase assay

Cells in a 24-well plate were transfected with siRNAs as above. On the fourth day, cells were transfected with SRF reporter p3DA.luc (8 ng) and reference reporter pTK-RL (20 ng), with or without normalization control SRF-VP16 (20 ng) by using JetPrime transfection reagent as above. Cells were maintained in DMEM containing 0.3% FBS for 24 h and stimulated if needed with serum (15%) or Cytochalasin D (2 μM). After 7 h of stimulation, cells were harvested and analysed with the Dual-Luciferase reporter assay system (Promega) and a luminometer, according to manufacturer’s instructions. For data analysis, the activity of firefly luciferase was normalized to the renilla luciferase activity and SRF-VP16 was set to 100.

### Expression and purification of Ddx19 protein

Recombinant mouse His-Ddx19 was produced by using the MultiBac baculovirus expression vector system. Briefly, pDEST10-mDDX19 plasmid was transformed into DH10MultiBac cells, and recombinant bacmids were isolated and the presence of the coding sequence of Ddx19 was confirmed by PCR. The bacmid was then transfected into IPLB-Sf21AE (Sf21) cells by using Fugene HD (Promega). Baculoviruses were amplified and expression of the protein conducted by infection 2 × 10^6^ Sf21 cells with the baculovirus stock at a multiplicity of infection 1. The cells were harvested after 72 h and the cell pellets were snap frozen and stored at −80 °C. The cell pellets were resuspended in lysis buffer (25 mM Tris-HCl pH 8.0, 300 mM NaCl, 0.5 mM EDTA, 2 mM B-mercaptoethanol, 0,2 % Igepal, 1 mM phenylmethyl sulphonyl fluoride) with protease inhibitors (Roche) and lysed with EmulsiFlex-C3 (AVESTIN) with gauge pressure ~15,000 psi for 10 min. Cells extracts were then clarified by centrifugation at 20,000*g* for 30 min and immediately processed to metal (Ni^2+^) affinity purification using Ni^2+^-NTA agarose beads. Beads were incubated with the extract for 2 h and washed three times with the buffer A (25 mM Tris-HCl pH 8.0, 300 mM NaCl, 0.5 mM EDTA) and then two times with buffer A with addition of 10 mM Imidazole. The protein was eluted from the beads with 500 mM Imidazole in lysis buffer. The protein was further purified with gel filtration column Superdex 200 (Superdex 200 HiLoad 16/60, Pharmacia), which was equilibrated with buffer B (25 mM Tris-HCl pH 7.5, 100 mM NaCl, 1 mM dithiothreitol (DTT), 10 % glycerol). Fractions were analysed by SDS–PAGE and those containing His-Ddx19 were concentrated and stored at −80 °C.

### GST pull-down assay

GST and GST fusion proteins from cleared *E. coli* (900 μl) lysates were immobilized on glutathione-sepharose beads (30 μl, GE Healthcare) for 2 h on rotation at +4 °C. Beads were washed three times with Binding buffer 1 (50 mM Tris-HCl pH 7.5, 300 mM NaCl, 10 mM EDTA, 1 mM phenylmethyl sulphonyl fluoride) and one time with Binding buffer 2 (10 mM Hepes pH 7.9, 1.5 mM MgCl_2_, 10 mM KCl, 0.1% Triton X-100) or 3 (5 mM Tris-HCl pH 7.5, 0.2 mM ATP, 0.2 mM DTT, 0.2 mM CaCl_2_, 100 mM NaCl). After washing, the beads were incubated with purified recombinant Ddx19 or with 500 μg cytoplasmic Hela or NIH 3T3 cell lysate, with or without 50 mM NaCl, 40 μm Ran-Q69L and energy mix (20 mM phosphocreatine, 1 mM GTP, 1 mM ATP, 50 μg ml^−1^ creatine phosphokinase) or 1–10 μm LatB-actin.

The binding was for 3 h on rotation at +4 °C and then the beads were washed three times with Binding buffer 2 or 3, and bound proteins were eluted with 1 × SDS–PAGE loading buffer (1% SDS, 62.5 mM Tris-HCl pH 6.8, 50 mM DTT, 10% glycerol, bromphenol blue). Samples were boiled for 5 min and the proteins were separated in 10–12% SDS–PAGE, stained with Coomassie brilliant blue or electroblotted to nitrocellulose membrane. The membrane was probed with Ddx19 or Ipoβ antibodies to assess their association with GST fusions.

His-tagged Ran Q69L was produced in bacteria. Ran Q69L in pQE plasmid was transformed into *E. coli* M15 cells and cultivated in Luria Bertani medium at +37 °C until the optical density at 600 nm was 0.6. Protein expression was induced with 0.5 mM isopropyl-β-D-1-thiogalactopyranoside and allowed to proceed for 3 h at +37 °C. Cells were collected by centrifugation and the cell pellet was resuspended in 1 × PBS with 1 mg ml^−1^ lysozyme (Sigma). Igepal CA-630 (Sigma) was added and the lysis was enhanced by sonication (6 × 20 s). The lysate was cleared by centrifugation and the His-tagged fusion proteins were bound to Ni-NTA Agarose beads (QIAGEN) for 2 h at +4 °C. Beads were washed two times with Wash buffer (50 mM Tris-HCl pH 7.5, 200 mM NaCl, 2 mM MgCl_2_, 10% glycerol). After two washes with 30 mM Imidazole in Wash buffer, the bound proteins were eluted with 500 mM Imidazole and 10 μM GTP in Wash buffer. The peak fractions were analysed on SDS–PAGE and the fractions that included the recombinant Ran Q69L protein were pooled and dialysed against 1 mM MgCl_2_, 10% glycerol and 1 mM DTT in 1 × PBS. After measuring the concentration, 1 mM GTP was added.

LatB-actin was prepared by incubating rabbit skeletal muscle actin with a tenfold molar excess of LatB (Calbiochem) overnight at +4 °C. Twenty times initiation buffer (2 M NaCl, 60 mM MgCl_2_ and 10 mM ATP) was added to polymerize uncomplexed actin and the filaments were removed by ultracentrifugation at 200,000 *g* for 15 min at +4 °C.

### Co-IP assays

NIH 3T3 cells (1 × 10^6^) were plated on 10 cm dishes and transfected with the appropriate HA-tagged constructs (3 μg each plasmid) using Jet Prime transfection reagent. Forty-eight hours later, cells were harvested in IP buffer (0.5% Triton X-100, 50 mM Tris-HCl pH 7.5, 150 mM NaCl) and the lysates were cleared by centrifugation. Cleared lysates were then subjected to EZview Red Anti-HA Affinity Gel (Sigma-Aldrich), incubated 3 h on rotation at +4 °C and washed three times with IP buffer without Triton X-100. Bound proteins were eluted with 1 × SDS–PAGE loading buffer, boiled for 5 min, separated in 10% SDS–PAGE and electroblotted to nitrocellulose membrane. The membrane was probed with indicated antibodies.

For co-IP with antibodies against endogenous proteins, NIH 3T3 cells on 10 cm plates were grown near confluency. Cells were harvested by trypsinization followed by gentle lysis (50 mM NaCl and 50 mM Hepes pH 7.4, 25 μg ml^−1^ Digitonin), to isolate the cytoplasmic fraction. Ddx19 antibody were added to the lysate (2 μg antibody per 1 mg protein) and incubated for 3 h on rotation at +4 °C. Magnetic DynaBeads (Invitrogen) were added and incubated 1 h on rotation at +4 °C. The beads were washed four times with buffer containing 50 mM NaCl and 50 mM Hepes pH 7.4 and the bound proteins were eluted with 1 × SDS–PAGE loading buffer and processed for western blotting with indicated antibodies.

### Real-time quantitative PCR

NIH 3T3 cells (60,000) were plated on 10 cm dishes and transfected with 10 nm siRNAs as above. On the fourth day, the media was changed to fresh growth media containing 0.3% FBS and re-transfected with siRNAs if needed. On the sixth day, cells were stimulated with 15% FBS for 90 min and total RNA was extracted using Nucleospin RNA II kit instructed by the manufacturer (Macherey-Nagel). One microgram of total RNA was used for complementary DNA synthesis using Thermo Scientific RT–PCR kit (Thermo Scienctific) and random primers. Quantitative PCR was carried out using the Bio-Rad CFX machine (Bio-Rad) and SYBR green qPCR reagent (Thermo Scientific). Gene specific primers are as follows:

Vinculin_for: 5′-CTTTGTGCAGGCAAGGAACG-3′

Vinculin_rev: 5′-GCTGCATTCTCCACTTTGGC-3′

Acta2_for: 5′-ACTGGGACGACATGGAAAAG-3′

Acta2_rev: 5′-GTTCAGTGGTGCCTCTGTCA-3′

Gapdh_for: 5′-TGCACCACCAACTGCTTAGC-3′

Gapdh_rev: 5′-GGCATGGACTGTGGTCATGAG-3′

Relative expression levels were calculated by the comparative *C*_T_ method, normalizing to the Gapdh cDNA: 2^−C^_T_ (target)/2^−C^_T_ (Gapdh).

### FRET/FLIM

FRET was measured by frequency domain FLIM. More information about the method can be found elsewhere^37^.

NIH3T3 cells (25,000) on a 6-well plate format were transfected with siRNAs as above. On the fourth day, cells were transfected with 100–200 ng of GFP, GFP-mCherry, MKL1-GFP, RPEL-GFP or mCherry-MKL1-GFP, with and without 300 ng mCherry or Ipoβ-mCherry constructs using JetPrime transfection reagent, as described before, and maintained for 18 h. Live cells were imaged in 37 °C under a CO_2_ hood. Zeiss Axio Observer Z1 microsope was used for the imaging. All images were acquired using a C-Apochromat × 63/1.2 numerical aperture water objective and 488 nm solid-state laser modulated at 50 MHz. The donor was excited using Semrock LF488-B filter set. Images were processed using Slidebook 5.5 and the lifetimes were calculated from selected regions of interests.

### Statistical analyses

Statistical analyses were performed in Excel or OriginPro 8.6. The data for SRF reporter activity, expression of SRF target genes and MKL1 nuclear localization in rescue experiment were analysed by two-tailed Student’s *t*-test, with two-sample unequal variance, because the data conformed to normal distribution. Significance was determined by *P*<0.05. Non-parametric Mann–Whitney test, with the significance level of 0.05, was applied to the FRET/FLIM data, because the data did not conform to normal distribution (Shapiro–Wilk).

Uncropped scans of all blots and gels are shown in Supplementary Fig. 8.

## Author contributions

M.K.V. supervised and designed the study. E.K.R. performed the experiments with valuable help from T.V., G.H. and S.K. M.K.V. and E.K.R. wrote the manuscript. R.T. contributed to study design and hosted M.K.V. for the initial stages of this project.

## Additional information

**How to cite this article**: Rajakylä, E. K. *et al.* RNA export factor Ddx19 is required for nuclear import of the SRF coactivator MKL1. *Nat. Commun.* 6:5978 doi: 10.1038/ncomms6978 (2015).

## Supplementary Material

Supplementary Information Supplementary Figures 1-8
