# Supplementary material for: RNA export factor Ddx19 is required for nuclear import of the SRF coactivator MKL1
Source: Nat Commun. 2015 Jan 14;6:5978. doi: 10.1038/ncomms6978 (PMC4309436; doi:10.1038/ncomms6978)
Supplement: Supplementary Information — Supplementary Figures 1-8 [file ncomms6978-s1.pdf]

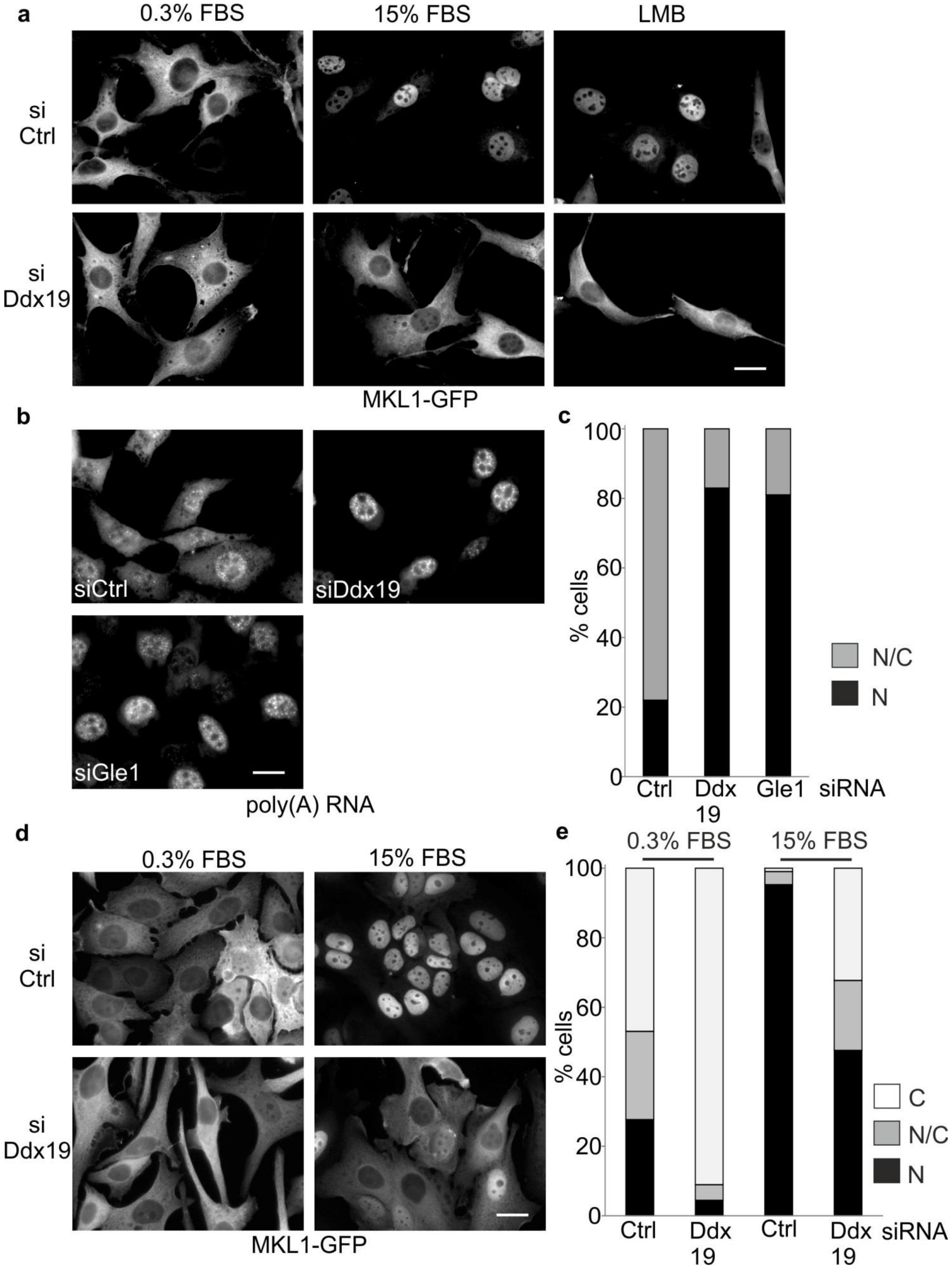
a
0.3% FBS
15% FBS
LMB
si Ctrl
si Ddx19
MKL1-GFP
b
siCtrl
siDdx19
siGle1
poly(A) RNA
c
% cells
0
20
40
60
80
100
Ctrl
Ddx 19
Gle1
siRNA
N/C
N
d
0.3% FBS
15% FBS
si Ctrl
si Ddx19
MKL1-GFP
e
0.3% FBS
15% FBS
% cells
0
20
40
60
80
100
Ctrl
Ddx 19
Ctrl
Ddx 19
siRNA
C
N/C
N

Supplementary Figure 1. Depletion of Ddx19 prevents nuclear accumulation of MKL1 in mouse and human cell lines. (a) Localization of MKL1-GFP in NIH 3T3 cells transfected with control or Ddx19 siRNAs (si) in serum starved (0.3% FBS) conditions or treated with serum (15% FBS) or Leptomycin B (LMB). **(b)** Localization of poly(A)RNA in cells treated with control, Ddx19 or Gle1 siRNAs. **(c)** Quantification of mRNA localization in NIH 3T3 cells transfected with indicated siRNAs. 100 cells per point. C/N, pancellular; N, nuclear. **(d)** Localization of MKL1-GFP in MCF7 cells treated with control or Ddx19 siRNAs at serum starved (0,3% FBS) or serum stimulated (15% FBS) conditions. **(e)** Quantification of MKL1-GFP localization in MCF7 cells as in (Fig. 1c), 100 cells per point. C, cytoplasmic; C/N, pancellular; N, nuclear Scale bars 20 μm.

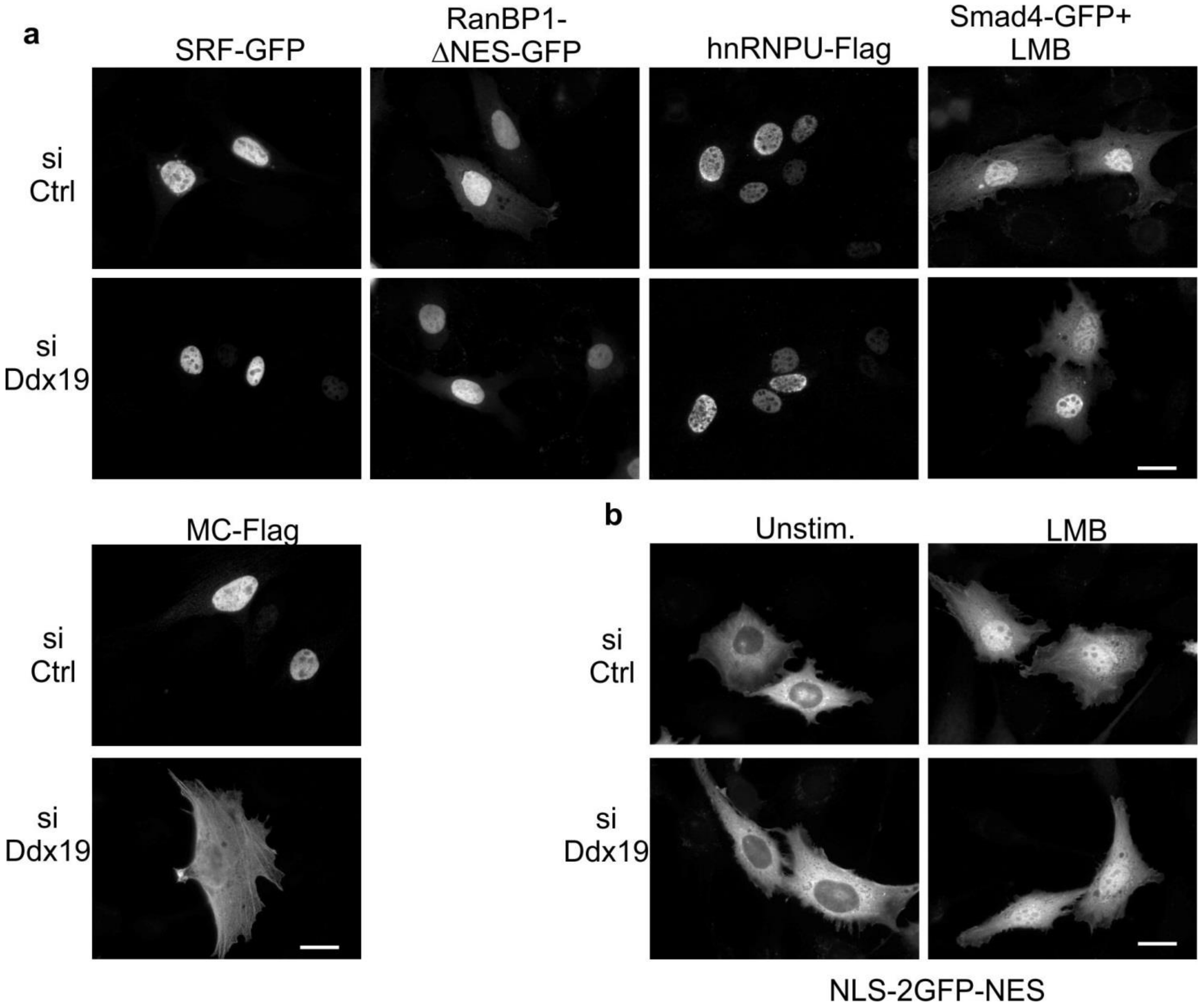

Supplementary Figure 2. Localization of control nuclear proteins in NIH 3T3 cells. (a) Localization of indicated constructs in NIH 3T3 cells transfected with control or Ddx19 siRNAs. SRF (serum response factor), RanBPI-ΔNES (Ran binding protein 1 lacking nuclear export signal), hnRNPU (heterogeneous nuclear ribonucleoprotein U), Smad4 + LMB (Smad4 stimulated with leptomycin B), MC (myocardin). **(b)** NLS-2GFP-NES localization in cells transfected with control or Ddx19 siRNAs and treated or not (Unstim) with Leptomycin B (LMB). Scale bars 20 µm.

```
CLUSTAL O(1.2.1) multiple sequence alignment

SP|Q61655|DD19A_MOUSE MATDSWALAVDEQEAAVKSMSSLQIKEEKVKADTNGVIKTSTTAEKTEEEEKEDRAAQSL 60
SP|P20449|DBP5_YEAST  --------MSDTKRDPADLLASLKIDNEKE--DTSEVSTKETVKSQPEKTADSIKPAEKL 50
                                * :.  .. ::**:*.:**   **. * ...*. .: *:  .. : *:.*

SP|Q61655|DD19A_MOUSE LN-------KLIRSNLV----DNTNQVEVLQRDPSSPLYSVKSFEELRLKPQLLQGVYAM 109
SP|P20449|DBP5_YEAST  VPKVEEKKTKQEDSNLISSEYEVKVKLADIQADPNSPLYSAKSFDELGLAPELLKGIYAM 110
                      :        *   ***:    : . ::  :* **.*****.***:** * *:**:*:***

SP|Q61655|DD19A_MOUSE GFNRPSKIQENALPMMLAEPPQNLIAQSQSGTGKTAAFVLAMLSRVEPADRYPQCLCLSP 169
SP|P20449|DBP5_YEAST  KFQKPSKIQERALPLLLHNPPRNMIAQSQSGTGKTAAFSLTMLTRVNPEDASPQAICLAP 170
                       *::******.***::* :**:*:************** *:**:**:* *  **.:**:*

SP|Q61655|DD19A_MOUSE TYELALQTGKVIEQMGKFHPELKLAYAVRGNKLERGQKVSEQIVIGTPGTVLDWCSKLKF 229
SP|P20449|DBP5_YEAST  SRELARQTLEVVQEMGKFTKITSQ--LIVPDSFEKNKQINAQVIVGTPGTVLDLMRR-KL 227
                      : *** ** :*:::****     .     :  :.:*: :::. *:::********   : *:
                                     E242Q             R261A/D
SP|Q61655|DD19A_MOUSE IDPKKIKVFVLDEADVMIATQGHQDQSIRIQRMLPRNCQMLLFSATFEDSVWKFAQKVVP 289
SP|P20449|DBP5_YEAST  MQLQKIKIFVLDEADNMLDQQGLGDQCIRVKRFLPKDTQLVLFSATFADAVRQYAKKIVP 287
                      :: :***:******* *:  **  **.**:*:**:: *::****** *:* ::*:*:**
                                     E240Q             R259A/D
SP|Q61655|DD19A_MOUSE DPNIIKLKREEETLDTIKQYYVLCNNREEKFQALCNLYGAITIAQAMIFCHTRKTASWLA 349
SP|P20449|DBP5_YEAST  NANTLELQTNEVNVDAIKQLYMDCKNEADKFDVLTELYGLMTIGSSIIFVATKKTANVLY 347
                      : * ::*: :* .:*:*** *: *:*. :**:.* :*** :**..::**  *:***. *
                                         R371G         V385N
SP|Q61655|DD19A_MOUSE AELSKEGHQVALLSGEMMVEQRAAVIERFREGKEKVLVTTNVCARGIDVEQVSVVINFDL 409
SP|P20449|DBP5_YEAST  GKLKSEGHEVSILHGDLQTQERDRLIDDFREGRSKVLITTNVLARGIDIPTVSMVVNYDL 407
                      .:*..***:*::* *:: .::* :*: ****:.***:**** *****:  **:*:*:**
                                  R428Q  R369G         V383N
SP|Q61655|DD19A_MOUSE PVDKDGNPDNETYLHRIGRTGRFGKRGLAVNMVDSKHSMNILNRIQEHFNK-KIERLDTD 468
SP|P20449|DBP5_YEAST  PTLANGQADPATYIHRIGRTGRFGRKGVAISFVHDKNSFNILSAIQKYFGDIEMTRVPTD 467
                      *.  :*: *  **:**********::*:*:.:*..*.*:***. **::* . :: *: **
                                      R426Q
SP|Q61655|DD19A_MOUSE DLDEIEKIAN----- 478
SP|P20449|DBP5_YEAST  DWDEVEKIVKKVLKD 482
                      * **:***.:
```

Supplementary Figure 3. Sequence alignment of mouse Ddx19 and yeast Dbp5 proteins with Clustal Omega program. Location of the used mutations in mouse and yeast proteins are indicated.

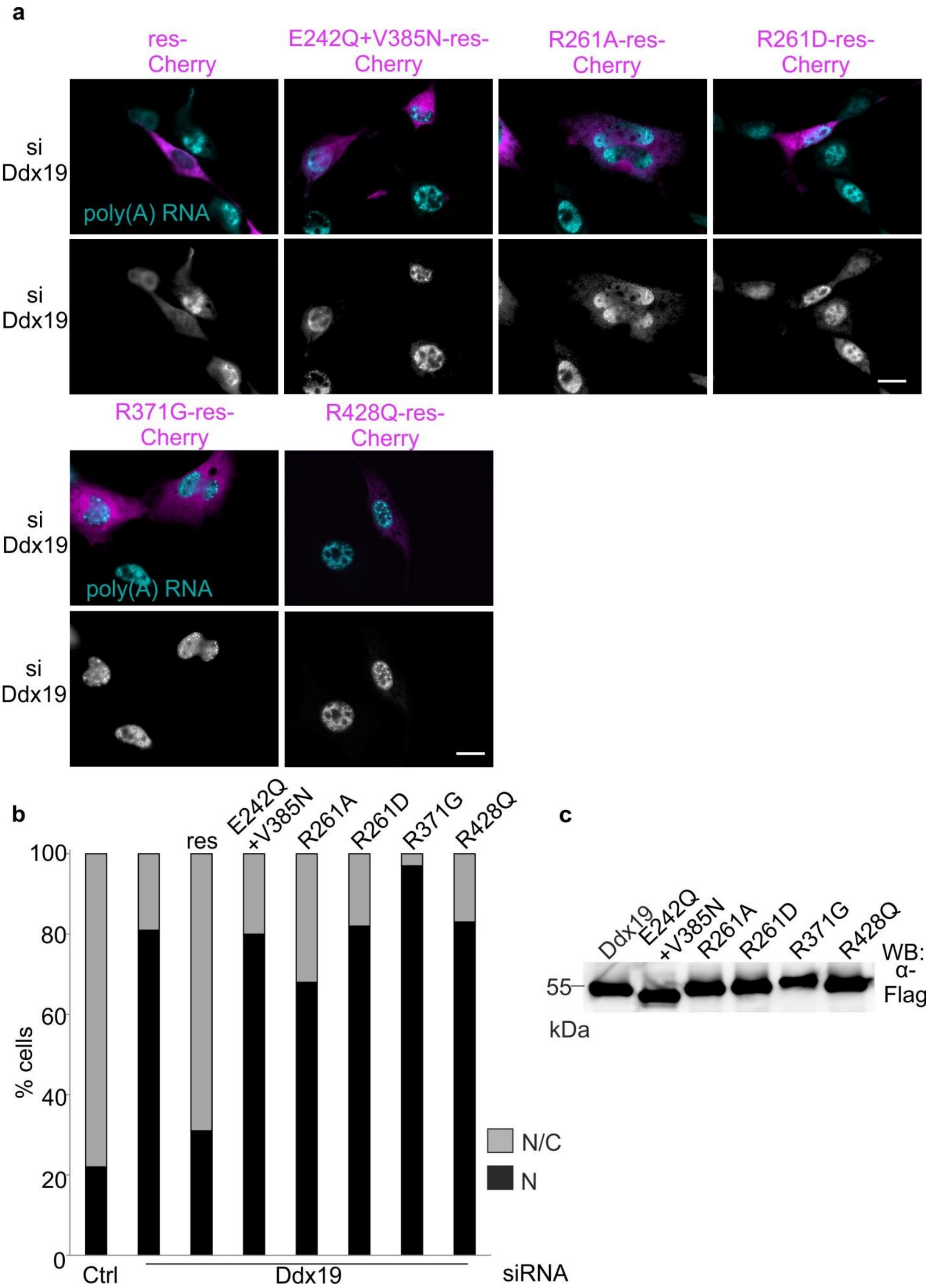
a
res-Cherry
E242Q+V385N-res-Cherry
R261A-res-Cherry
R261D-res-Cherry
si Ddx19
poly(A) RNA
si Ddx19
R371G-res-Cherry
R428Q-res-Cherry
si Ddx19
poly(A) RNA
si Ddx19
b
res
E242Q +V385N
R261A
R261D
R371G
R428Q
100
80
60
40
20
0
% cells
N/C
N
Ctrl
Ddx19
siRNA
c
Ddx19
E242Q +V385N
R261A
R261D
R371G
R428Q
WB: α-Flag
55
kDa

Supplementary Figure 4. FISH was performed to test the functionality of the Ddx19 mutant constructs. (a) mRNA distribution by FISH (cyan) in cells depleted of Ddx19 (siDdx19) and transfected with the indicated siRNA resistant Ddx19 constructs (magenta). The used constructs are defective for helicase activity (E242Q + V385N), Nup214- (R261A and R261D) or RNA-binding (R371G and R428Q). Scale bars 20 µm. **(b)** Quantification of mRNA localization in NIH 3T3 cells transfected with indicated siRNAs and Ddx19 mutants. 100 cells per point. C/N, pancellular; N, nuclear. **(c)** NIH 3T3 cells were transfected with Flag-tagged Ddx19 constructs and their expression was detected by Flag-antibody.

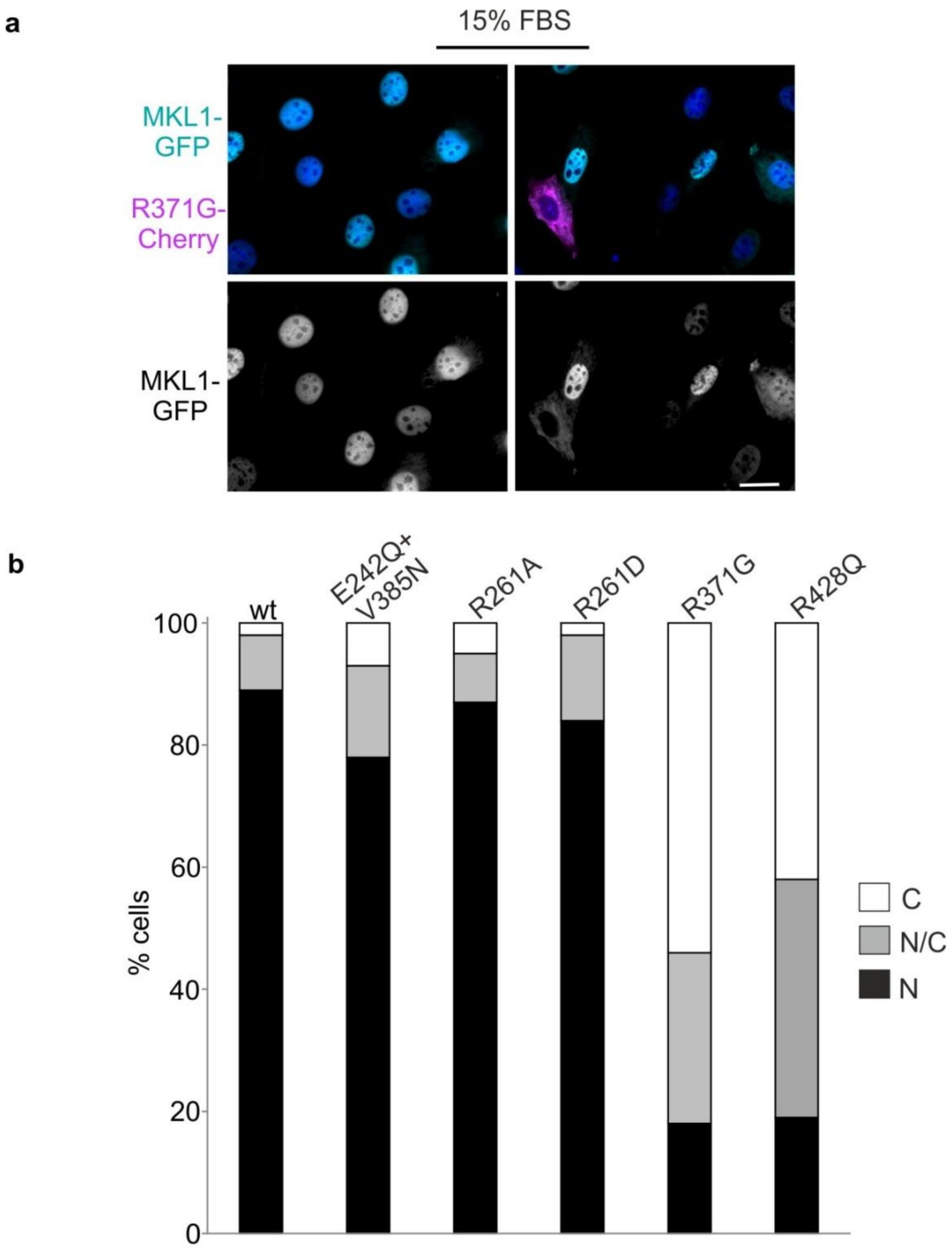

Supplementary Figure 5. Dominant negative phenotypes of the Ddx19-R371G and Ddx19-R428Q mutants. (a) In serum stimulated conditions (15% FBS), MKL1 (cyan) fails to accumulate in the nucleus in cells, which express Ddx19-R371G mutant (magenta). Scale bar 20 µm. **(b)** Quantification of MKL1-GFP localization in serum stimulated R332 cells transfected with wild-type Ddx19 (wt) or indicated point mutants. 100 cells per point. C, cytoplasmic; C/N, pancellular; N, nuclear.

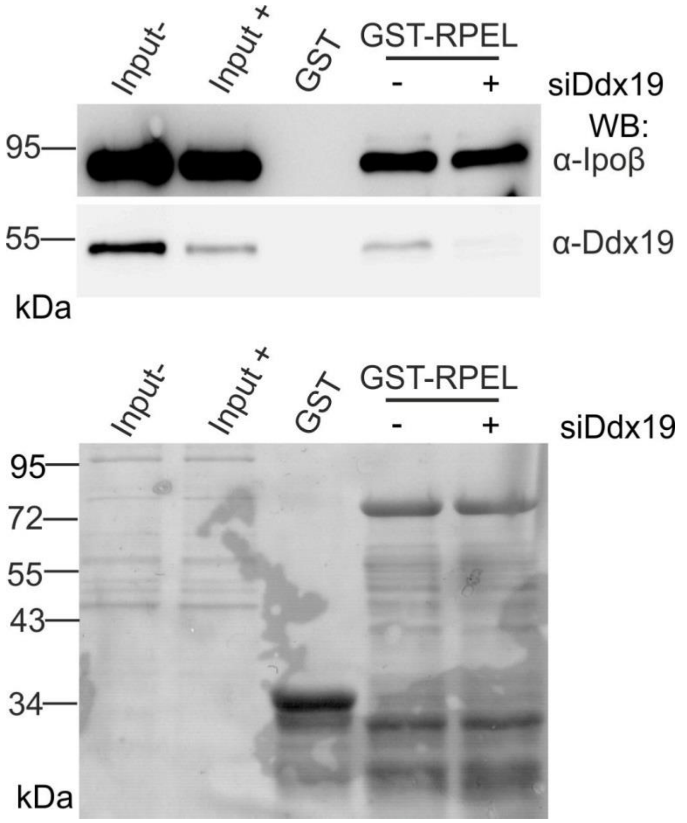

Supplementary Figure 6. Ipoβ binding to MKL1 RPEL domain does not depend on Ddx19. Recruitment of Ipoβ and Ddx19 from Ddx19 depleted NIH 3T3 cytoplasmic lysates by GST-RPEL. (-), NIH 3T3 cytoplasmic lysate transfected with control siRNAs; (+), Ddx19-depleted NIH 3T3 cytoplasmic lysate. Ponceau-stained full membrane (below).

a

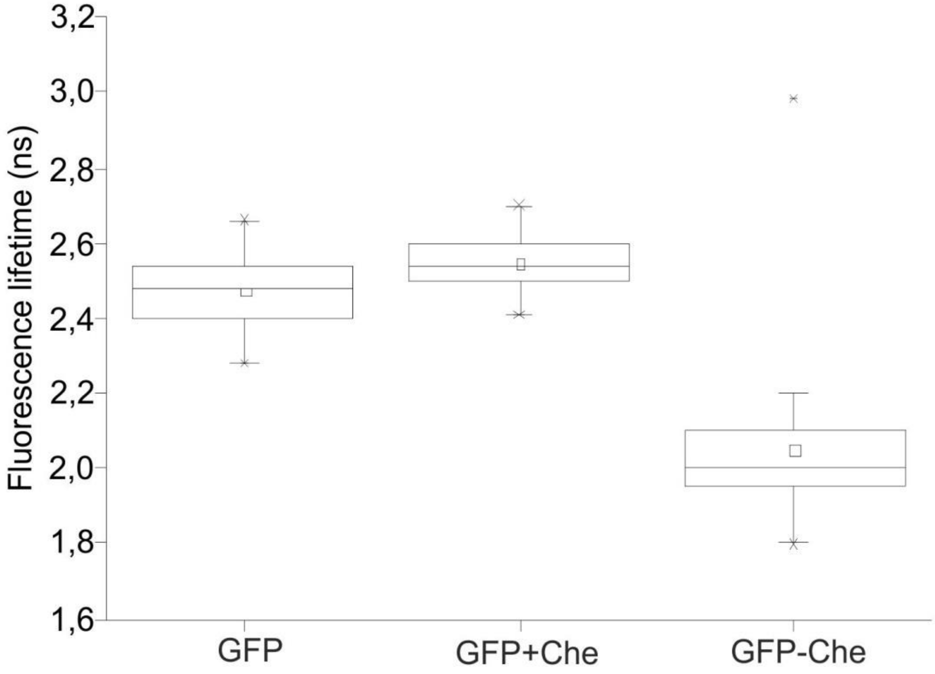

Fluorescence lifetime (ns)
3,2
3,0
2,8
2,6
2,4
2,2
2,0
1,8
1,6
GFP
GFP+Che
GFP-Che

b

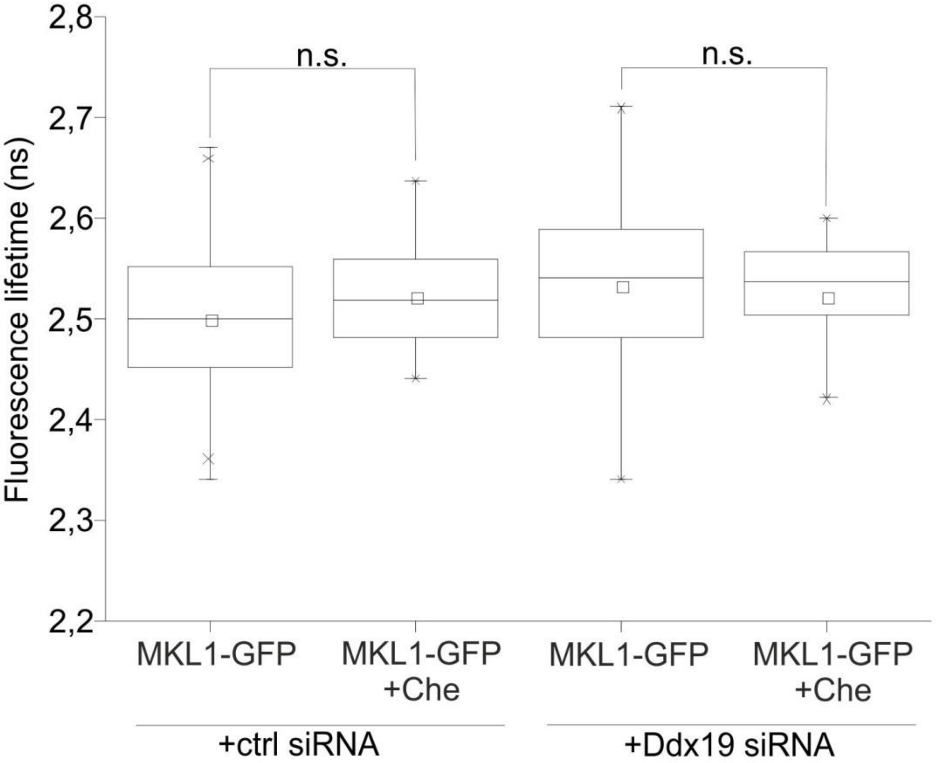

Fluorescence lifetime (ns)
2,8
2,7
2,6
2,5
2,4
2,3
2,2
n.s.
n.s.
MKL1-GFP
MKL1-GFP +Che
MKL1-GFP
MKL1-GFP +Che
+ctrl siRNA
+Ddx19 siRNA

Supplementary Figure 7. FRET/FLIM controls. (a) Fluorescence lifetimes of GFP, GFP co-transfected with free mCherry (GFP + Che) as a negative control and GFP containing C-terminal mCherry tandem construct (GFP-Che) as a positive control. **(b)** Fluorescence lifetimes of MKL1-GFP with and without free mCherry in control (ctrl) and Ddx19 depleted cells. Fluorescence lifetimes are shown as box-and-whisker plots, where the upper and lower limits of the box corresponds to the 75th and 25th percentiles, the median is indicated by the central bar and the whiskers indicate the minimum and maximum values. 24-111 cells per condition. Statistics with Mann-Whitney test using 0.05 confidence level. n.s. not significant differences. U values: ctrl siRNA MKL1-GFP vs. MKL1-GFP + Che 0,16883; Ddx19 siRNA MKL1-GFP vs. MKL1-GFP +Che 0,4218.

Fig. 1a

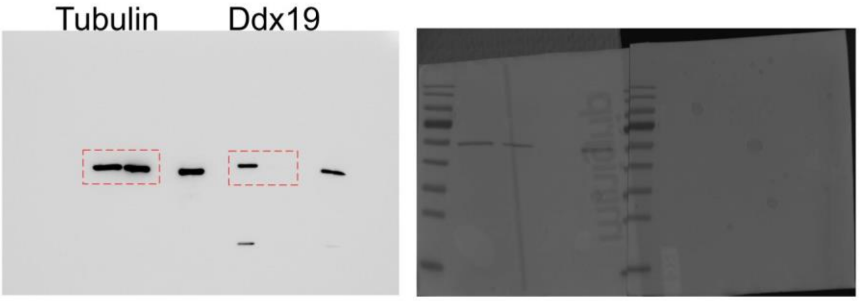

Fig. 4a

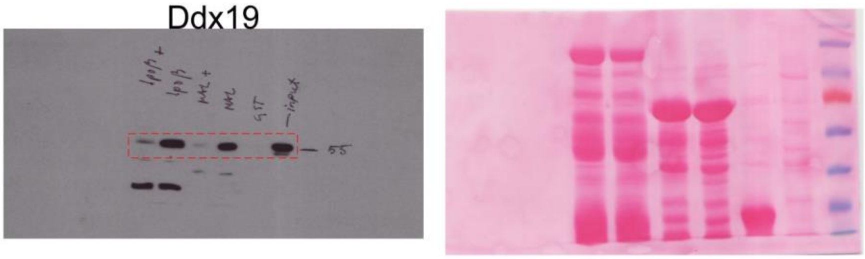

Fig. 4b

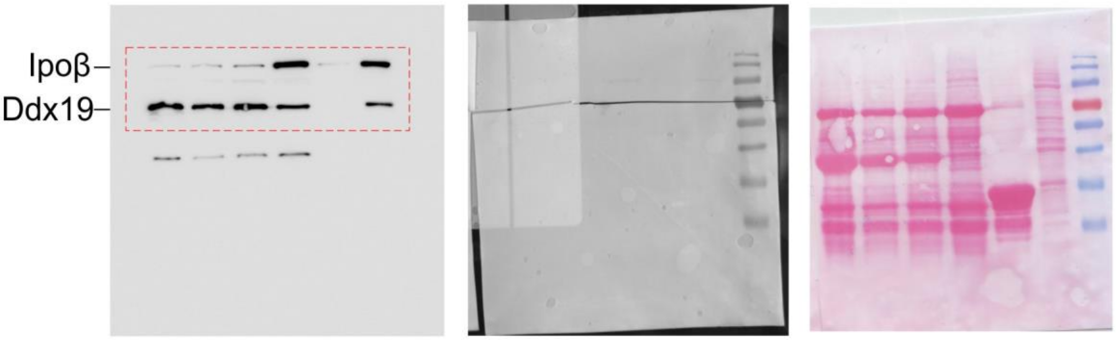

Fig. 5a

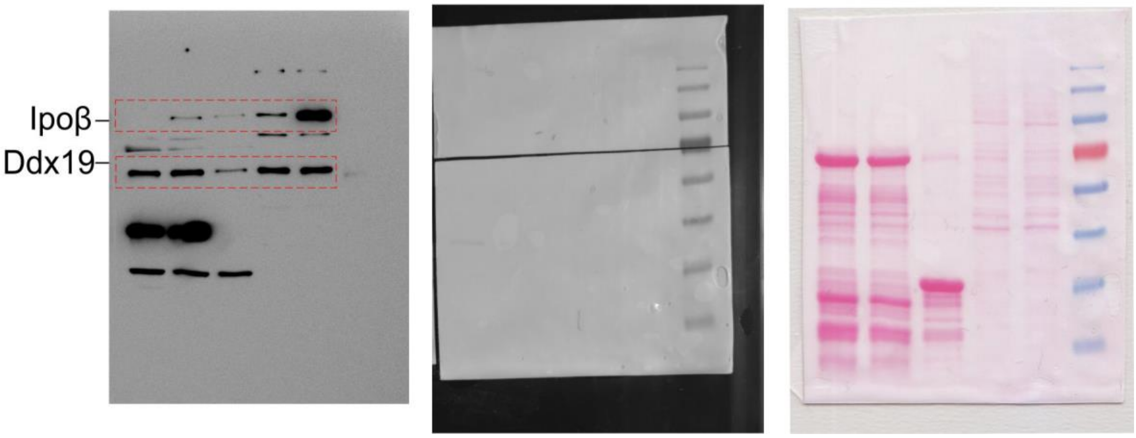

Fig. 5b

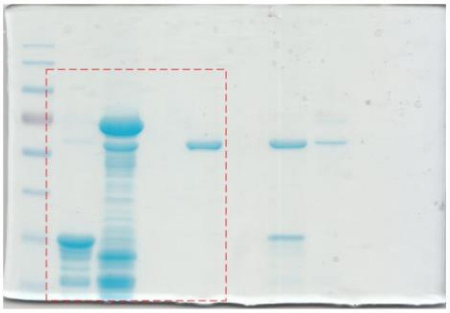

Fig. 7a

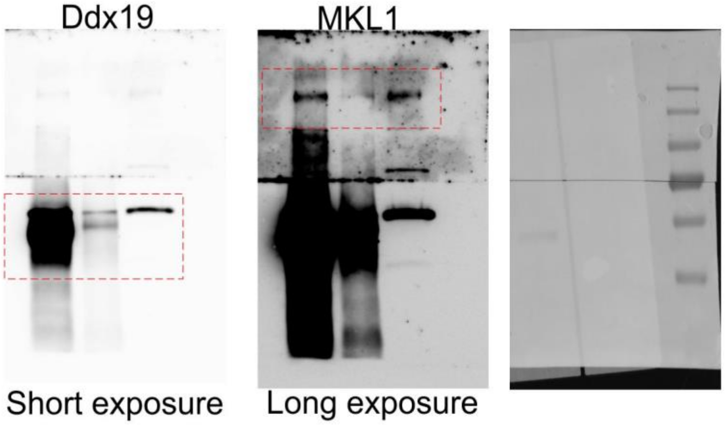

Fig. 7b

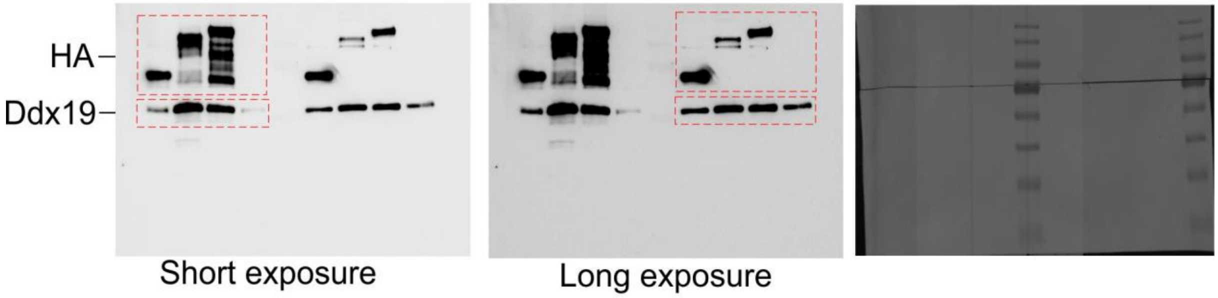

Supplementary Fig. 4c

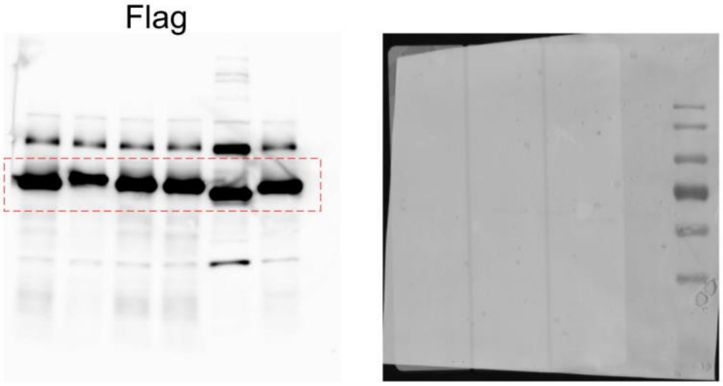

Supplementary Fig. 6

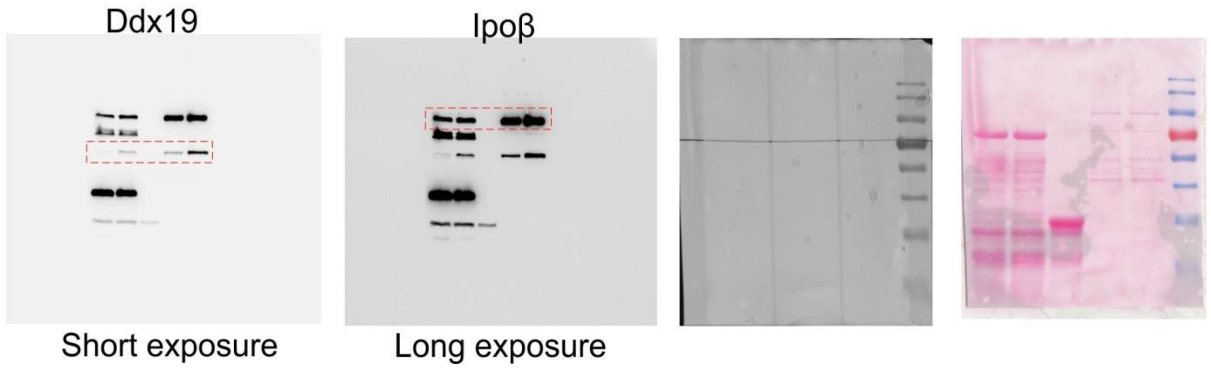

Supplementary Figure 8. Uncropped scans of all Western blots and a gel with the molecular weight markers.
